# Integrative taxonomy of the genus *Pseudoacanthocephalus* (Acanthocephala: Echinorhynchida) in China, with the description of two new species and the characterization of the mitochondrial genomes of *Pseudoacanthocephalus sichuanensis* sp. n. and *Pseudoacanthocephalus nguyenthileae*

**DOI:** 10.1186/s13071-024-06528-7

**Published:** 2024-12-27

**Authors:** Cui-Hong Zhao, Rui-Jia Yang, Si-Si Ru, Hui-Xia Chen, Dai-Xuan Li, Liang Li

**Affiliations:** 1 Hebei Collaborative Innovation Center for Eco-Environment, Hebei Key Laboratory of Animal Physiology, Biochemistry and Molecular Biology, College of Life Sciences, Hebei Normal University, Shijiazhuang, 050024 Hebei Province People’s Republic of China; 2 Hebei Research Center of the Basic Discipline Cell Biology, Ministry of Education Key Laboratory of Molecular and Cellular Biology, Shijiazhuang, 050024 Hebei Province People’s Republic of China

**Keywords:** Acanthocephala, Pseudoacanthocephalidae, Integrated taxonomy, Species delimitation, Mitochondrial genome, Cryptic species

## Abstract

**Background:**

Acanthocephalans (thorny headed worms) of the genus *Pseudoacanthocephalus* mainly parasitize amphibians and reptiles across the globe. Some species of the genus *Pseudoacanthocephalus* also can accidentally infect human and cause human acanthocephaliasis. Current knowledge of the species composition of the genus *Pseudoacanthocephalus* from amphibians and reptiles in China is incomplete. An insufficiency of genetic data on species of the genus *Pseudoacanthocephalus*, including the complete mitochondrial genomes, has limited the use of molecular-based methods to better define the taxonomy and phylogeny of the genus *Pseudoacanthocephalus*. A more rigorous molecular phylogeny with broader representatives of the genus *Pseudoacanthocephalus* is required to further clarify the systematic status of the family Pseudoacanthocephalidae.

**Methods:**

Many specimens of the genus *Pseudoacanthocephalus* collected from toads and frogs in China were identified to species level using integrated morphological methods (light and scanning electron microscopy) and molecular approaches (sequencing different nuclear and mitochondrial genetic markers). The Assemble Species by Automatic Partitioning (ASAP) and Bayesian inference (BI) methods were applied for species delimitation. The complete mitochondrial genomes of two *Pseudoacanthocephalus* species were also sequenced and annotated to enrich the body of mitogenomic data on acanthocephalans. Additionally, phylogenetic analyses based on the amino acid sequences of 12 protein-coding genes (PCGs) of mitochondrial genomes of acanthocephalans using maximum likelihood (ML) and BI were performed to further investigate the phylogenetic position of the family Pseudoacanthocephalidae in the order Echinorhynchida.

**Results:**

Three *Pseudoacanthocephalus* species, including *P. sichuanensis* sp. n., *P. previatesticulus* sp. n. and *P. nguyenthileae* were described. The results of ASAP and BI analyses based on the cytochrome* c* oxidase subunit 1 and subunit 2 (*cox1*, *cox2*) and 12S ribosomal RNA (12S) sequences supported the separation of *P. sichuanensis* and *P. previatesticulus* from the congeneric species. The results of BI inference using the internal transcribed spacer (ITS), *cox1*, *cox2* and 12S sequence data indicated that *P. sichuanensis* and *P. nguyenthileae* have a closer relationship than *P. previatesticulus* and *P. bufonis* in *Pseudoacanthocephalus*. The complete mitogenomes of *P. sichuanensis* and *P. nguyenthileae* have 15,812 and 13,701 bp, respectively, with both including 36 genes and two non-coding regions. Phylogenetic results based on mitogenomic data demonstrated that the two families Pseudoacanthocephalidae and Arhythmacanthidae have a sister relationship in the order Echinorhynchida.

**Conclusions:**

Two new species of the genus *Pseudoacanthocephalus*, namely *P. sichuanensis* sp. n. and *P. previatesticulus* sp. n., were identified based on integrated evidence. This is the first report of *P. nguyenthileae* in China. A revised key for the species of the genus *Pseudoacanthocephalus* was provided. Molecular analyses revealed that the mitochondrial *cox1*, *cox2* and 12S genes as genetic markers seem to be more suitable for species delimitation of *Pseudoacanthocephalus* than the nuclear ITS region. BI results suggested a close affinity between *P. sichuanensis* and *P. nguyenthileae*. The mitochondrial genomic data of *P. sichuanensis* and *P. nguyenthileae* are provided for the first time. Mitogenomic phylogenetic results further confirmed the validity of the family Pseudoacanthocephalidae.

**Graphical Abstract:**

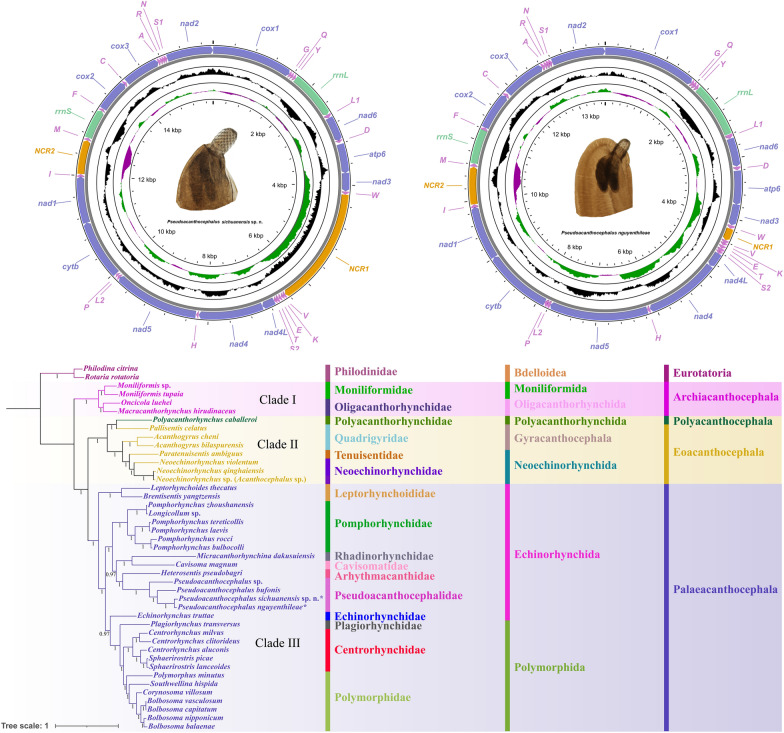

**Supplementary Information:**

The online version contains supplementary material available at 10.1186/s13071-024-06528-7.

## Background

The monotypic family Pseudoacanthocephalidae (type genus *Pseudoacanthocephalus*) is a common group of acanthocephalans occurring in the alimentary canal of amphibians and reptiles [1–5]. Petrochenko [6] erected the family Pseudoacanthocephalidae, but the validity of this family was subsequently rejected by the authors of a number of other studies [7–9]. More recently molecular phylogenetic results support the resurrection of the family Pseudoacanthocephalidae [10–13], but only one to two species of the Pseudoacanthocephalidae were included in these molecular phylogenies. A more rigorous molecular phylogenetic study that includes a broader representation of Pseudoacanthocephalidae is needed to investigate in detail the phylogenetic position of the Pseudoacanthocephalidae.

To date, 21 nominal species in the genus *Pseudoacanthocephalus* have been reported worldwide [4, 14, 15]. Among these, only five species have been recorded in China, including *P. bufonis*, *P. bufonicola*, *P. elongatus*, *P. lucidus* and *P. reesei* [3, 16–18]. The current taxonomy of the genus *Pseudoacanthocephalus* is still mainly based on traditional taxonomic methods. Although molecular-based approaches are effective and practical for discriminating morphologically similar species, detecting cryptic species and delimiting the phenotypic variation of acanthocephalans [19–22], molecular-based taxonomy of the genus *Pseudoacanthocephalus* remains in the initial stage owing to the insufficiency of available molecular databases. So far, only six species of the genus *Pseudoacanthocephalus* have been sequenced for their genetic data, namely *P. nickoli*, *P. bufonis*, *P. nguyenthileae*, *P. smalesae*, *P. toshimai* and *P. lucidus* [4, 5, 12, 23]. Among these, only *P. bufonis* and *Pseudoacanthocephalus* sp. have been reported for the mitochondrial genomic data [12].

In the present study, large numbers of acanthocephalans collected from amphibians in China were identified to species level based on integrated methods (including light and scanning electron microscopy, and sequencing some different nuclear and mitochondrial markers). The Assemble Species by Automatic Partitioning (ASAP) and Bayesian inference (BI) methods were also applied for species delimitation. The complete mitochondrial genomes of two *Pseudoacanthocephalus* species were sequenced and annotated to enrich the mitogenomic data of acanthocephalans. Additionally, phylogenetic analyses based on the amino acid sequences of 12 protein-coding genes (PCGs) of the acanthocephalan mitogenomes using the maximum likelihood (ML) and BI methods were performed to further determine the phylogenetic position of the family Pseudoacanthocephalidae in the order Echinorhynchida.

## Methods

### Acanthocephalan collection

A total of 100 acanthocephalan specimens were isolated from the intestines of *Bufo gargarizans* (Anura: Bufonidae) in Sichuan Province, 27 acanthocephalan specimens were collected from the intestine of *Bufo melanostictus* in Hainan Province and four acanthocephalan specimens obtained from the intestines of *Quasipaa exilispinosa* (Anura: Dicroglossidae) in the Guangxi Zhuang Autonomous Region, China. Specimens were washed in saline, then placed in 80% ethanol until studied.

### Morphological observation

For the light microscopical studies, acanthocephalans were first cleared in lactophenol. Drawings were made with the aid of a Nikon microscope drawing attachment (Nikon Corp., Tokyo, China). For scanning electron microscopy (SEM), specimens were post-fixed in 1% OsO4, dehydrated via an ethanol series and acetone and then critical point dried. The samples were coated with gold and examined using a Hitachi S-4800 scanning electron microscope at an accelerating voltage of 20 kV (Hitachi Ltd., Tokyo, Japan). Measurements (the range, followed by the mean in parentheses) were provided in micrometers unless otherwise stated.

### Molecular procedures

The genomic DNA of acanthocephalans was extracted using a Column Genomic DNA Isolation Kit (Shanghai Sangon, Shangai, China) according to the manufacturer's instructions. The primers and cycling conditions used for amplifying the target sequences of 18S ribosomal RNA (rRNA), 28S (large ribosomal unit), internal transcribed spacer (ITS), cytochrome* c* oxidase subunit 1 and 2 (*co*x*1*, *cox2*) and the small subunit rRNA sequence (12S), are provided in Additional file 4: Table S1. PCR products were purified, sequenced and analyzed according to methods reported previously [11–13].

### Species delimitation

The ASAP [24] and BI inference analyses were used to delimit *Pseudoacanthocephalus* species based on the partial ITS, *cox1*, *cox2* and 12S sequence data, respectively. The ASAP analyses were performed using the ASAP online server (https://bioinfo.mnhn.fr/abi/public/asap) under the Kimura (K80) ts/tv model. The optimal result was considered according the lowest score and special recommendation by ASAP. The BI analyses were run using MrBayes 3.2.7 [25] under the HKY + F + G4 for ITS, *cox1* and 12S, and HKY + F + I for *cox2* (two parallel runs, 2,000,000 generations). *Pomphorhynchus tereticollis* was treated as the outgroup. The ingroup for the genus* Pseudoacanthocephalus* included *P. toshimai*, *P. lucidus*, *P. smalesae*, *P. bufonis*, *P. nickoli* and the material of *Pseudoacanthocephalus* spp. in the present study.

### Assembly and annotation of mitogenomes

A total of 30 Gb of gene library data of each acanthocephalan species was obtained by the Illumina NovaSeq 6000 platform using the Pair-End 150 sequencing method (Novogene, Tianjin, China). The methods and procedures used for assembly and annotation of the complete acanthocephalan mitochondrial genomes in the present study have been published previously [12, 13]. Various software programs or tools were used, including GetOrganelle v1.7.2a [26], MitoS web server (http://mitos2.bioinf.uni-leipzig.de/index.py), MitoZ v2.4 [27], ORF finder web server (https://www.ncbi.nlm.nih.gov/orffinder/), ViennaRNA module [28], MitoS2 [29], RNAstructure v6.3 [30] and Codon Adaptation Index (CAI) [31]. MitoZ v2.4 was employed to visualize and depict gene element features of mitochondrial genomes [27].

### Mitogenomic phylogenetic analyses

Phylogenetic analyses were performed based on the amino acid sequences of 12 PCGs of acanthocephalan mitogenomes, using the ML and BI methods, respectively. *Philodina citrina* and *Rotaria rotatoria* (Bdelloidea: Philodinidae) were treated as the outgroup. The ingroup included 41 acanthocephalan species (detailed information provided in Additional file 5: Table S2. Genes were aligned separately using MAFFT v7.313 under the iterative refinement method of E-INS-I [32]. Ambiguous sites and poorly aligned positions were eliminated using BMGE v1.12 (*m* = BLOSUM90, *h* = 0.5) [33]. The aligned and eliminated sequences were concatenated into a matrix by PhyloSuite v1.2.2 [34]. The partitioning schemes and the optimal amino acid substitution model selected for each combination of partition for the ML and BI inference are provided in Additional file 6: Table S3. Reliabilities tested for ML inference, Bayesian information criterion (BIC) analysis and the bootstrap (BS) values and Bayesian posterior probabilities (BPP) values for consideration as constituting strong or moderate branch support are according to previous studies [11, 13].

## Results

### Species identification

Among the observed acanthocephalans, 27 individuals from *B. melanostictus* in Hainan Province were identified as *P. nguyenthileae*. However, the other acanthocephalans obtained from *B. gargarizans* in Sichuan Province and *Q. exilispinosa* in Guangxi Zhuang Autonomous Region, represented two undescribed species. The morphological characteristics of these two species acanthocephalans obtained in this study are described in the following sections.

#### *Pseudoacanthocephalus sichuanensis* sp. n. (Figs. 1, 2, 3)

**Fig. 1:**
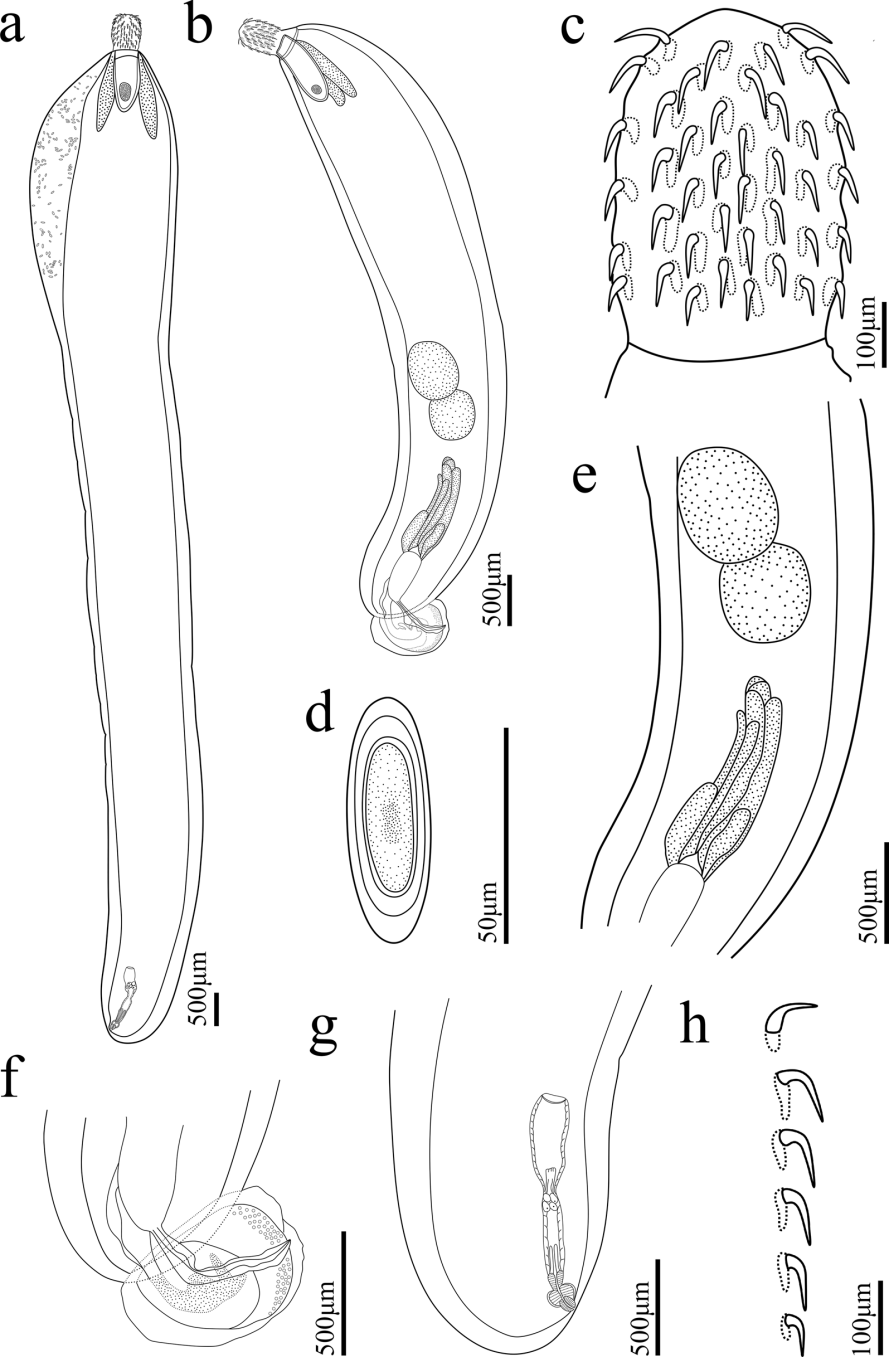
*Pseudoacanthocephalus sichuanensis* sp. n. collected from *Bufo gargarizans* in China.** a** Mature female,** b** mature male,** c** proboscis of male,** d** egg,** e** testes and cement glands,** f** copulatory bursa,** g** posterior end of female,** h** proboscis hooks of female

**Fig. 2:**
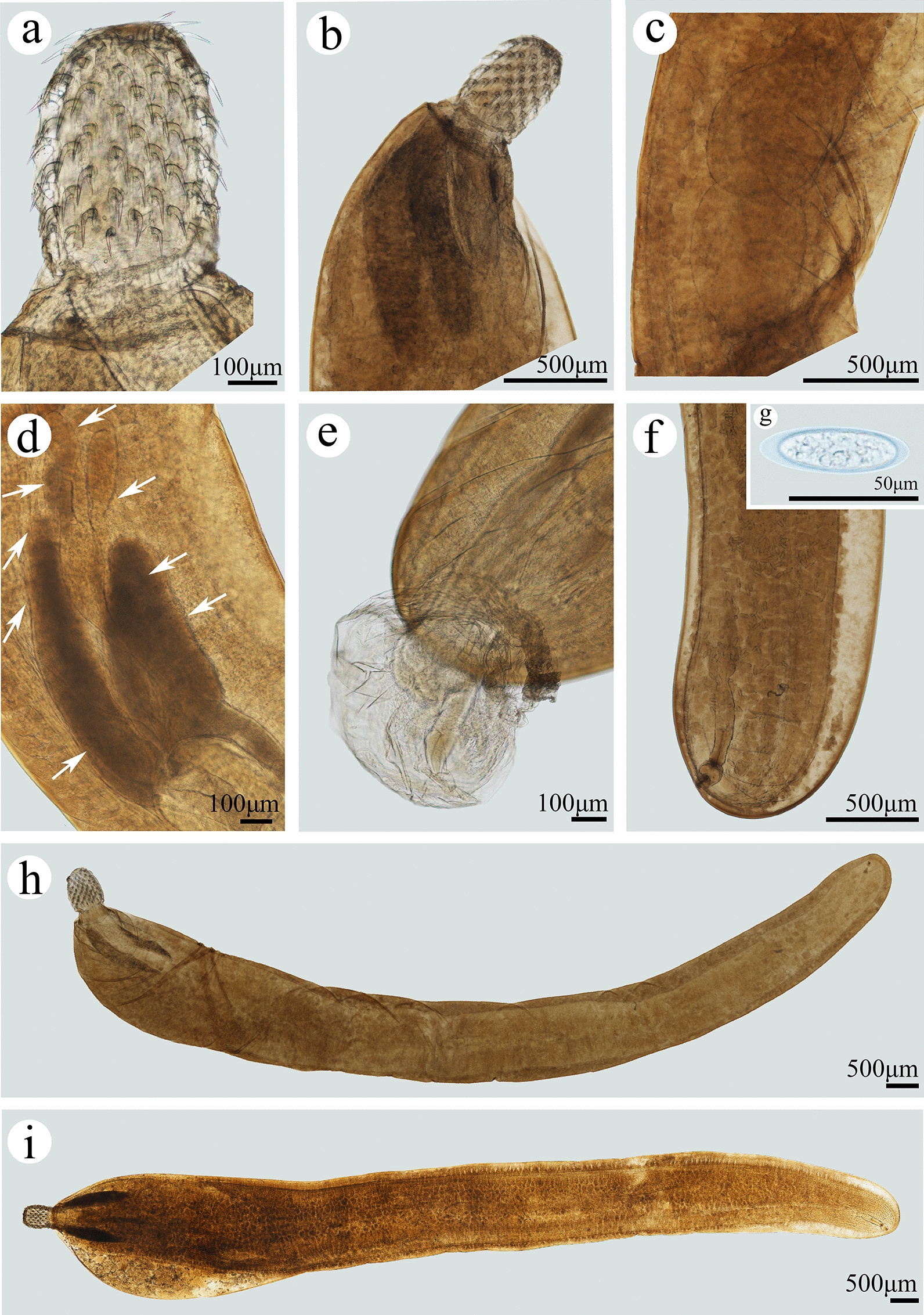
Photomicrographs of *Pseudoacanthocephalus sichuanensis* sp. n. collected from *Bufo gargarizans* in China.** a** Proboscis of male,** b** anterior part of male,** c** testes,** d** cement glands (arrowed),** e** copulatory bursa,** f** posterior end of female,** g** egg,** h**,** i** mature female

**Fig. 3:**
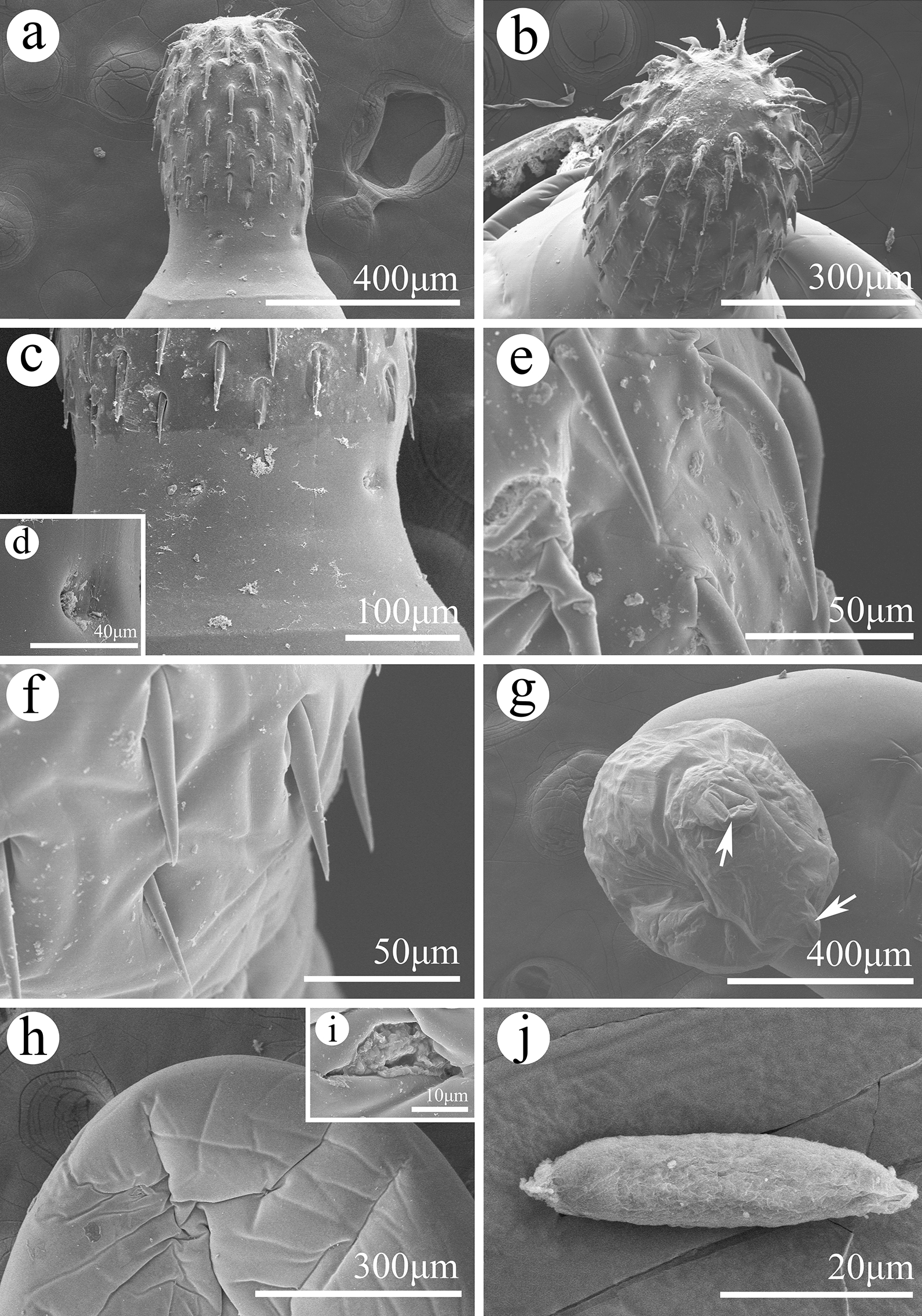
Scanning electron micrographs of *Pseudoacanthocephalus sichuanensis* sp. n. collected from *Bufo gargarizans* in China.** a** Proboscis of male, lateral view,** b** proboscis of male, apical view,** c** neck of male,** d** sensory pit in neck,** e** magnified image of middle proboscis hooks of female,** f** magnified image of posterior proboscis hooks of female,** g** copulatory bursa (2 auriform protrusions indicated by arrows),** h** magnified image of sensory papillae on copulatory bursa,** i** magnified image of genital pore,** j** egg

*Type-host*: *Bufo gargarizans* Cantor (Anura: Bufonidae).

*Type-locality*: Beichuan, Mianyang City, Sichuan Province.

*Site in host*: Intestine.

*Prevalence and intensity of infections*: Two out of four individuals of *B. gargarizans* both infected with 100 acanthocephalans.

*Type specimens*: Holotype, 1 male (HBNU–A–A2024001ZL); allotype, 1 female (HBNU–A–A2024002ZL); paratype: 30 males and 30 females (HBNU–A–A2024003ZL); deposited in the College of Life Sciences, Hebei Normal University, Hebei Province, China.

*Representative DNA sequences*: Representative genetic sequences were deposited in the National Center for Biotechnology Information (NCBI) database (http://www.ncbi.nlm.nih.gov) under the accession numbers: PP454074, PP454075 (18S); PP471560, PP471561 (28S); PP466922, PP466923 (ITS); PP469226, PP469227 (*cox1*); PP486179, PP486180 (*cox2*); PP471567, PP471568 (12S); PP476191 (mitogenome).

*ZooBank registration*: The Life Science Identifier (LSID) of the article is urn: lsid: zoobank.org: pub: D8FF19E0-AE31-4055-A498-597F5EB9718B. The LSID for the new name *Pseudoacanthocephalus sichuanensis* is urn: lsid: zoobank.org: act: D1BD3790-4DDB-4D2C-9EB7-57325F4B542D.

*Etymology*: The specific name refers to its geographic origin (Sichuan Province).

**Description:** Trunk medium-sized, smooth, more or less cylindrical; widest near anterior 1/6–1/5 of trunk, gradually tapering toward posterior end (Figs. 1a, b, 2h, i). Female larger than male, anterior part of trunk (tegument) in some individuals of female distinctly thickened dorsally (possible fixation artefact) (Figs. 1a, 2i). Proboscis almost cylindrical, armed with 18–20 longitudinal rows of 4–6 (generally 5) rooted hooks each (Figs. 1c, 2a, b, 3a, b). Hook size increases from apex to middle part of proboscis, distinctly decreases towards base (Figs. 1h, 2a, 3a, c, f). Proboscis receptacle double-walled, cerebral ganglion at posterior of proboscis receptacle (Fig. 1a, b). Neck short, with several cupped pores (Fig. 3a, c, d). Lemnisci slightly unequal, longer than proboscis receptacle (Figs. 1a, b, 2b). Genital pore subterminal in male and female (Figs. 1f, g, 2e, f).

##### Male (based on 10 mature specimens)

Trunk 6.78–8.61 (7.64) mm long, maximum width 1.44–1.71 (1.58) mm. Proboscis 400–460 (452) long, 270–350 (328) wide. Length of anterior proboscis hooks 50–88 (75), middle proboscis hooks 70–88 (78), basal proboscis hooks 50–70 (60). Length of roots of anterior hook 23–36 (33), roots of middle hook 43–57 (47), roots of basal hook 36–50 (41). Proboscis receptacle 0.73–1.10 (0.88) mm long, 244–366 (305) wide. Neck 100–200 (138) long, 250–580 wide (448). Longer lemniscus 1.12–1.34 (1.16) mm long, 244–390 (286) wide; shorter lemniscus 0.88–1.22 (1.05) mm long, 220–390 (266) wide. Testes two, ovoid, postequatorial, tandem and contiguous, anterior testis 0.75–1.02 (0.83) mm long, 480–659 (569) wide; posterior testis 550–976 (756) long, 512–660 (586) wide (Figs. 1e, 2c). Cement glands six long tubular, two short clavate; six long cement glands 1.34–1.71 (1.54) mm long, two short cement glands 512–756 (640) long (Figs. 1b, e, 2d). Copulatory bursa everted, with two auriform protrusions surrounded by numbers of sensory papillae (Figs. 1b, f, 2e, 3g), 360–840 (600) long, 650–900 (762) wide.

##### Female (based on 10 mature specimens)

 Trunk 9.76–18.0 ( 14.3) mm long, maximum width 1.37–1.90 (1.77) mm. Proboscis 550–650 (598) long, 420–554 (460) wide. Length of anterior proboscis hooks 53–105 (82), middle proboscis hooks 73–105 (86), basal proboscis hooks 58–75 (68). Length of roots of anterior hook 30–59 (47), roots of middle hook 53–59 (55), roots of basal hook 35–53 (45). Proboscis receptacle 460–950 (835) long, 250–535 (346) wide. Neck 130–220 (181) long, 450–663 (544) wide. Longer lemniscus 1.02–1.60 (1.39) mm long, 120–341 (232) wide; shorter lemniscus 0.65–1.55 (1.26) mm long, 150–280 (230) wide. Uterine bell 248–550 (440) long, uterus 300–560 (430) long, vagina 150–360 (230) long (Figs. 1g, 2f). Eggs elliptoid, 45–83 (59) × 13–28 (18) in size, without distinct polar prolongation of fertilization membrane (Figs. 1d, 2g, 3i).

**Remarks**

In the genus *Pseudoacanthocephalus*, *P. sichuanensis* sp. n. is similar to the following species by having a cylindrical proboscis with 16–20 longitudinal rows of four to five hooks each, including *P. bufonis* (Shipley 1903), *P. caspanensis*, *P. nguyenthileae*, *P. nickoli*, *P. toshimai*, *P. lutzi* and *P. goodmani* [1–5, 23, 35]. *Pseudoacanthocephalus sichuanensis* differs from *P. bufonis*, *P. caspanensis*, *P. goodmani*, *P. toshimai* and *P. lutzi* by having eight cement glands (vs no more than 6 cement glands in the latter five species). The new species can be easily distinguished from *P. nguyenthileae* and *P. nickoli* by the basal proboscis hooks being distinctly smaller than that of the middle part of the proboscis (proboscis hooks not decreasing in size towards base in the latter two species). *Pseudoacanthocephalus nickoli* is also different from *P. sichuanensis* by the trunk shape (trunk narrowed in the middle region and widened again towards posterior end in *P. nickoli *vs trunk widest near anterior 1/6–1/5 of trunk, gradually tapering toward posterior end in the new species). In the genus *Pseudoacanthocephalus*, only *P. coniformis*, *P. rauschi* and *P. nguyenthileae* have eight cement glands in the male [1, 14, 15], which is similar to that of the new species. However, *P. sichuanensis* with a proboscis possessing 18–20 longitudinal hook rows distinctly differ from the latter three species (vs proboscis possessing only 13 longitudinal hook rows in *P. coniformis*, 16–18 longitudinal hook rows in *P. nguyenthileae* and 16–18 longitudinal hook rows in *P. rauschi*). Moreover, molecular analysis showed high level of nucleotide divergence between *P. sichuanensis* and the above-mentioned similar congeneric species in the ITS sequences (14.0% for *P. toshimai*, 7.60–7.61% for *P. bufonis*, 16.2% for *P. nickoli*), *cox1* (28.5–29.1% for *P. toshima*, 11.3–12.1% for *P. nguyenthileae*, 26.2–26.6% for *P. bufonis*) and *cox2* (12.4% for *P. nguyenthileae*, 32.7% for *P. bufonis*).

Among the five species of *Pseudoacanthocephalus* recorded in China [3, 16, 17], *P. elongatus*, with a remarkably elongated proboscis and each longitudinal row with 13–15 hooks, can be easily differentiated from the new species. *Pseudoacanthocephalus reesei* and *P. lucidus* differ from *P. sichuanensis* by having a proboscis with 12–15 (usually 14) longitudinal rows and six cement glands (vs proboscis with 18–20 longitudinal rows and 8 cement glands in *P. sichuanensis*). *Pseudoacanthocephalus bufonicola* is different from *P. sichuanensis* by having a distinctly smaller trunk (4.00–4.50 mm), a much longer proboscis (0.77 mm) and each longitudinal row of proboscis with six to seven hooks in the male (vs trunk 7.27–8.61 mm, proboscis 0.40–0.46 mm, each longitudinal row of proboscis with 4–5 hooks in male in the new species).

#### *Pseudoacanthocephalus previatesticulus* sp. n. (Figs. 4, 5, 6)

**Fig. 4:**
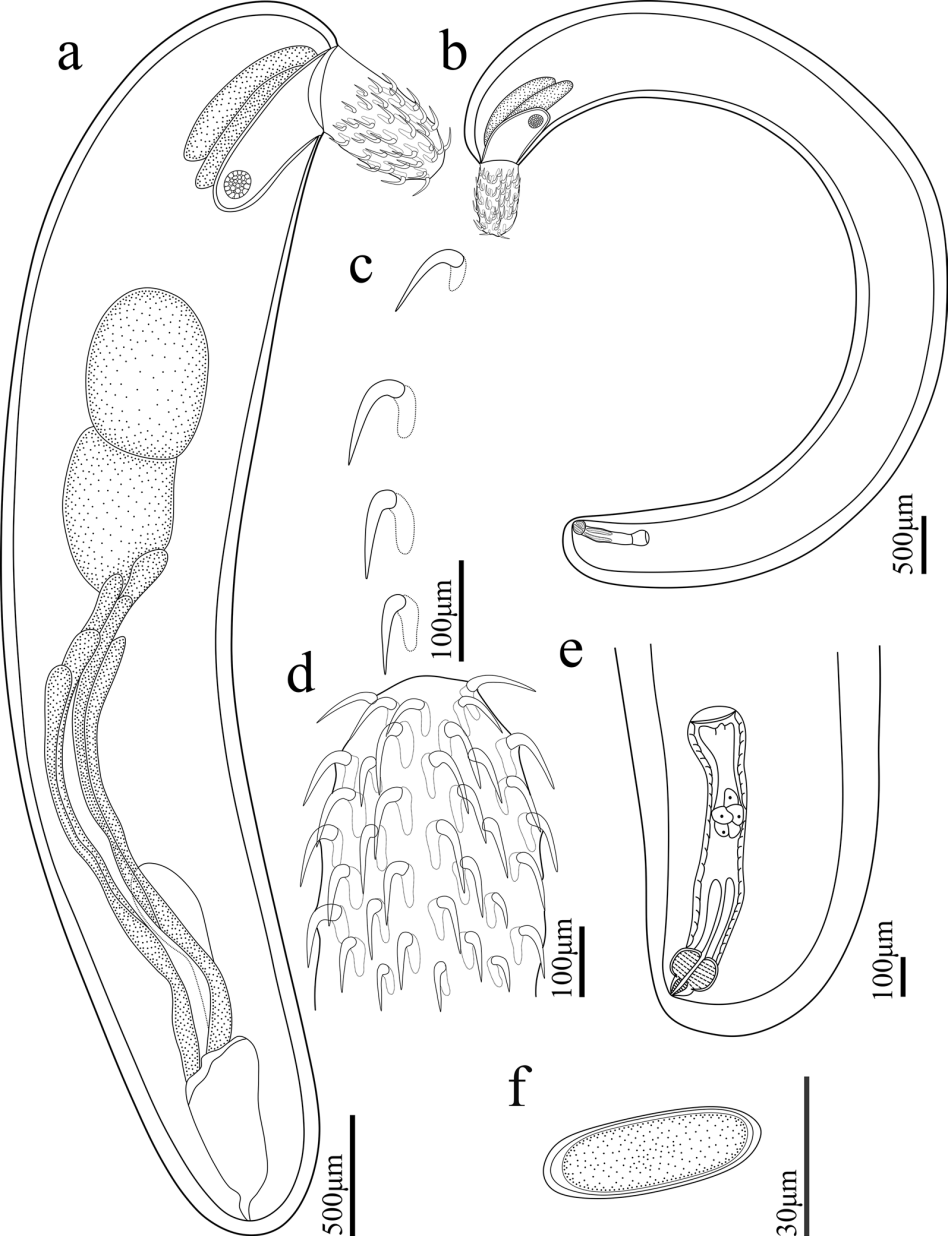
*Pseudoacanthocephalus previatesticulus* sp. n. collected from *Quasipaa exilispinosa* in China.** a** Mature male,** b** mature female,** c** proboscis hooks of male,** d** proboscis of male,** e** posterior end of female,** f** egg

**Fig. 5:**
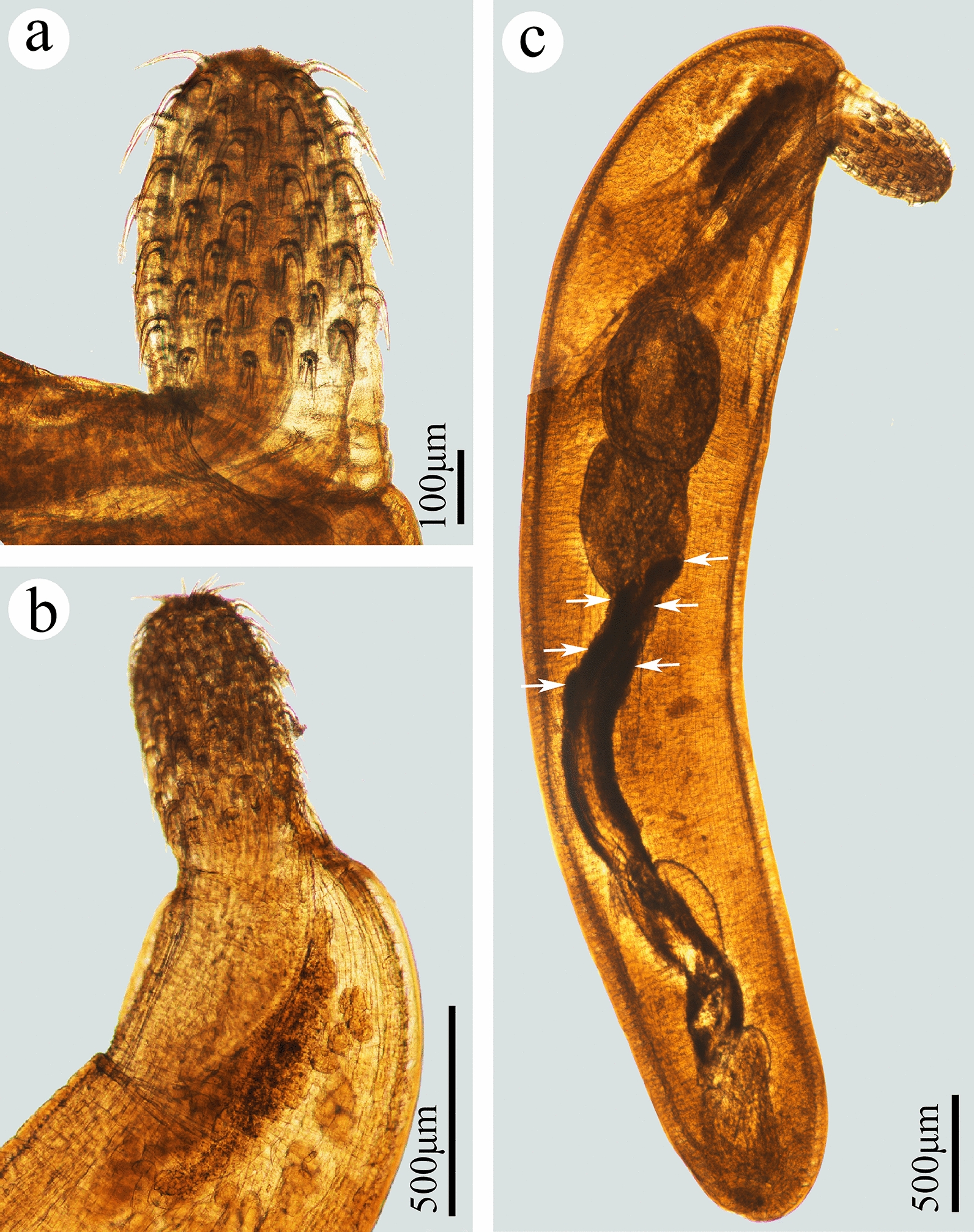
Photomicrographs of *Pseudoacanthocephalus previatesticulus* sp. n. collected from *Quasipaa exilispinosa* in China.** a** Proboscis of male,** b** anterior part of female,** c** mature male (cement glands arrowed)

**Fig. 6:**
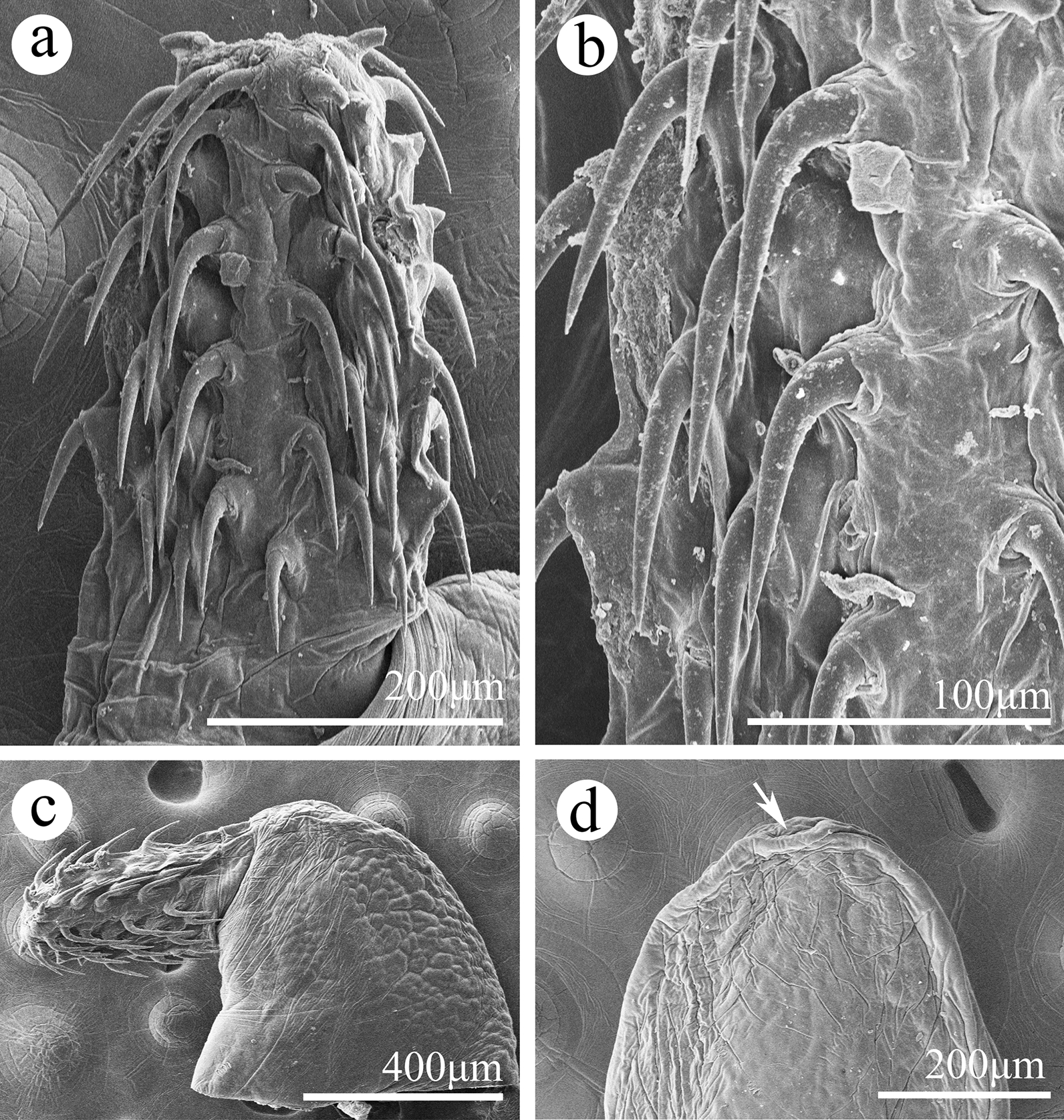
Scanning electron micrographs of *Pseudoacanthocephalus previatesticulus* sp. n. collected from *Quasipaa exilispinosa* in China.** a** Proboscis of male,** b** magnified image of proboscis hooks,** c** anterior part of male,** d** posterior end of male (gonopore arrowed)

*Type-host*: *Quasipaa exilispinosa* (Liu & Hu) (Anura: Dicroglossidae).

*Type-locality*: Dayaoshan Mountain, Jinxiu City, Guangxi Zhuang Autonomous Region.

*Site in host*: Intestine.

*Prevalence and intensity of infections*: One out of three individuals of *Q. exilispinosa* infected with intensity of 4 acanthocephalans.

*Type specimens*: Holotype, 1 male (HBNU–A–A2024004ZL); allotype, 1 female (HBNU–A–A2024005ZL); paratype: 1 male (HBNU–A–A2024006ZL); deposited in the College of Life Sciences, Hebei Normal University, Hebei Province, China.

*Representative DNA sequences*: Representative genetic data were deposited in the NCBI database (http://www.ncbi.nlm.nih.gov) under the accession numbers: PP455352 (18S); PP471559 (28S); PP466921 (ITS); PP481149 (*cox1*); PP486181 (*cox2*); PP471564 (12S).

*ZooBank registration*: The LSID of the article is urn: lsid: zoobank.org: pub: D8FF19E0-AE31-4055-A498-597F5EB9718B. The LSID for the new name *Pseudoacanthocephalus previatesticulus* is urn: lsid: zoobank.org: act: B4553D37-BA38-4864-B8A4-4740AE82EA4F.

*Etymology*: The specific name refers to the particular location of the testes in male.

**Description**: Trunk small, smooth, more or less cylindrical; widest near anterior 1/4–1/3 of trunk, gradually tapering toward posterior end (Figs. 4a, b, 5c). Female distinctly larger than male. Proboscis nearly cylindrical, armed with 16–18 longitudinal rows of four to five (generally 5) rooted hooks each (Figs. 4d, 5a, b, 6a, b). Hook size increases from apex to middle part of proboscis, then decreases towards base (Figs. 4c, d, 5a, 6a–c). Proboscis receptacle double-walled, cerebral ganglion at posterior of proboscis receptacle (Figs. 4a, b, 5b, c). Neck short. Lemnisci slightly subequal, as long as proboscis receptacle or longer than proboscis receptacle (Figs. 4a, b, 5b, c). Genital pore subterminal or nearly terminal in male and female (Figs. 4e, 5c, 6d).

##### Male (based on 2 mature specimens)

Trunk 4.85–5.33 (5.15) mm long, maximum width 1.05–1.08 (1.06) mm. Proboscis 366–594 (495) long, 287–366 (333) wide. Length of anterior proboscis hooks 63–108 (102), middle proboscis hooks 100–115 (108), basal proboscis hooks 75–95 (86). Length of roots of anterior hook 39–61 (50), roots of middle hook 61–74 (68), roots of basal hook 43–57 (52). Proboscis receptacle 594–693 (644) long, 228–356 (294) wide. Neck 99–154 (127) long, 307–346 wide (327). Lemnisci 594–891 (658) long, 139–248 (190) wide. Testes two, ovoid, pre-equatorial, anterior testis 594–772 (660) long, 495–634 (548) wide; posterior testis 633–693 (673) long, 455–653 (535) wide (Figs. 4a, 5c). Cement glands six, very long, tubular; 1.33–2.35 (1.96) long (Figs. 4a, 5c). Copulatory bursa 554–644 (614) long, 297–446 (396) wide.

##### Female (based on 1 mature specimen)

 Trunk 8.45 mm long, maximum width 0.93 mm. Proboscis 644 long, 396 wide. Length of anterior proboscis hooks 45–65 (58), middle proboscis hooks 58–65 (61), basal proboscis hooks 45–53 (48). Length of roots of anterior hook 34–56 (47), roots of middle hook 45–62 (55), roots of basal hook 34–56 (48). Proboscis receptacle 594 long, 386 wide. Neck 59 long, 446 wide. Lemnisci 743 long, 198 wide. Uterine bell 159 long, uterus 470 long, vagina 129 long (Fig. 4e). Eggs elliptoid, 37–44 (40) × 12–15 (12) in size, without distinct polar prolongation of fertilization membrane (Fig. 4f).

**Remarks**

In the genus *Pseudoacanthocephalus*, *P. previatesticulus* with six long tubular cement glands and pre-equatorial testes, can be easily distinguished from all of the above-mentioned similar congeneric species *P. bufonis*, *P. caspanensis*, *P. nguyenthileae*, *P. nickoli*, *P. toshimai*, *P. lutzi*, *P. goodmani* and *P. sichuanensis*, with a cylindrical proboscis possessing 16–20 longitudinal rows of four to five hooks each [1–5, 23, 35], and all of the *Pseudoacanthocephalus* spp. recorded in China, including *P. bufonicola*, *P. elongatus*, *P. lucidus* and *P. reesei* (vs all of these species with relative short cement glands and post-equatorial testes) [3, 16, 17]. In the genus *Pseudoacanthocephalus*, only *P. smalesae* has pre-equatorial testes. However, the proboscis of *P. smalesae* is armed with only 12–13 longitudinal rows of hooks (vs 16–18 longitudinal rows of hooks in *P. previatesticulus*).

#### *Pseudoacanthocephalus nguyenthileae* Amin, Ha & Heckmann, 2008 (Figs. 7, 8; Table 1)

**Fig. 7:**
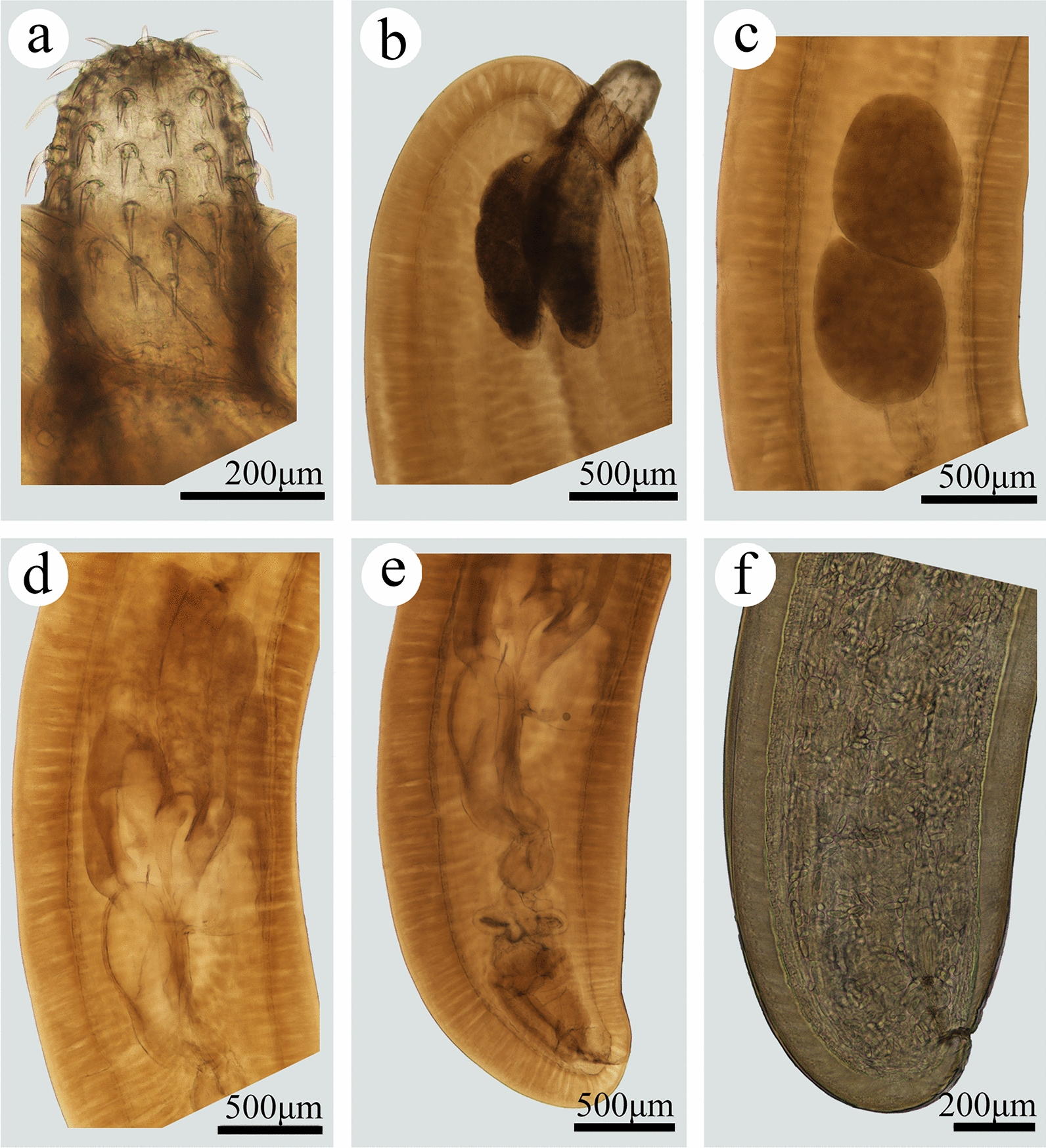
Photomicrographs of *Pseudoacanthocephalus nguyenthileae* collected from *Bufo melanostictus* in China.** a** Proboscis of male,** b** anterior part of male,** c** testes,** d** cement glands,** e** posterior end of male,** f** posterior end of female

**Fig. 8:**
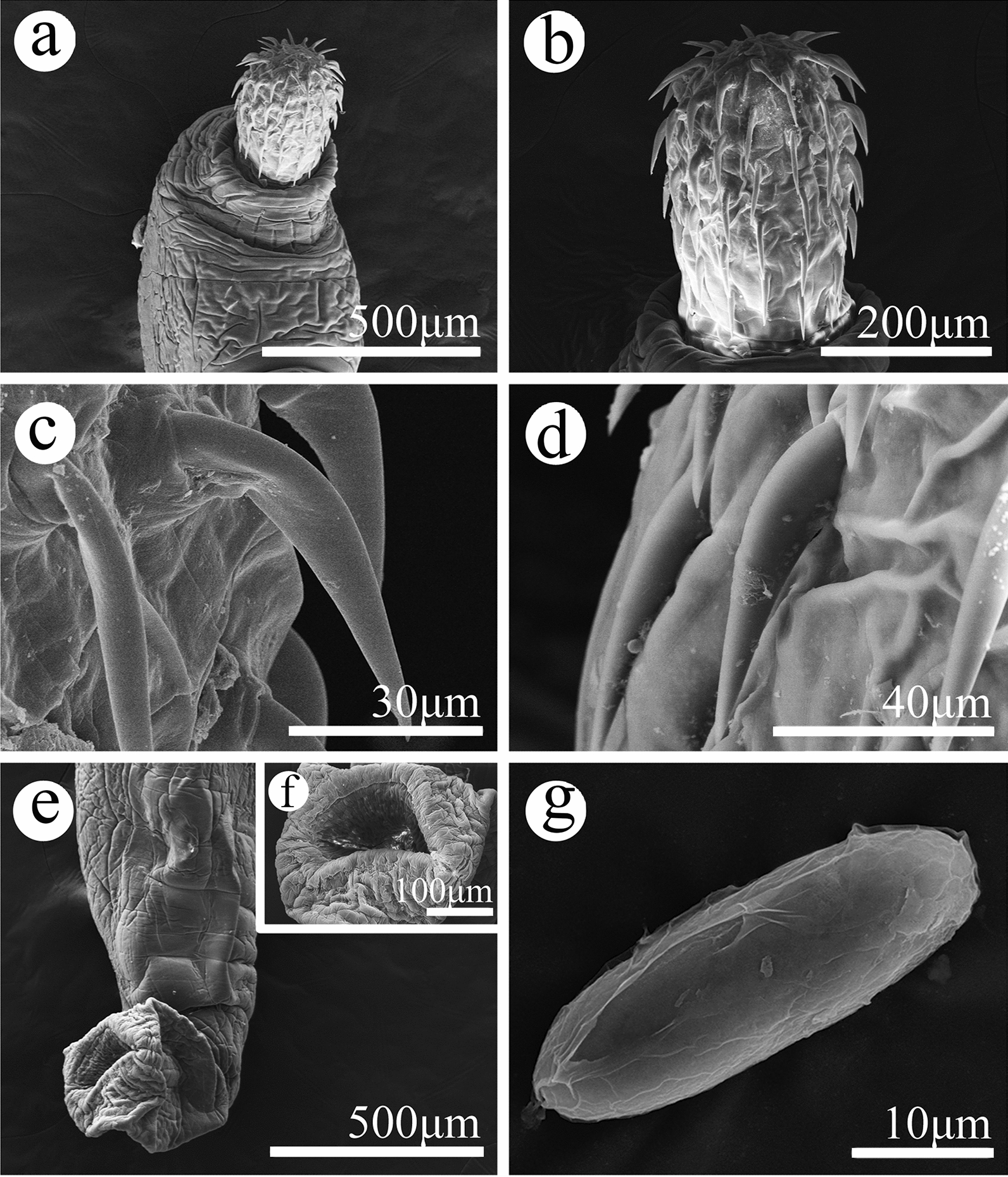
Scanning electron micrographs of *Pseudoacanthocephalus nguyenthileae* collected from *Bufo melanostictus* in China.** a** Anterior part of male,** b** proboscis of male,;** c** magnified image of anterior proboscis hooks,** d** magnified image of posterior proboscis hooks,** e** posterior part of male,** f** magnified image of copulatory bursa,** g** egg

**Table 1:** Comparative morphometric data for *Pseudoacanthocephalus nguyenthileae*

Parameters	Present study		Amin et al. [1]	
	Male	Female	Male	Female
TL	5.10–10.3	8.00–20.0	4.12–8.62	8.00–28.0
TW	0.90–1.60	0.98–1.73	0.67–1.55	0.95–2.40
PS	0.36–0.48 × 0.23–0.32	0.23–0.56 × 0.30–0.38	0.36–0.54 × 0.21–0.38	0.35–0.70 × 0.30–0.43
NHR	16–18	16–18	15–19	15–19
NHPR	5	5	5	5–6
NS	0.07–0.13 × 0.28–0.34	0.06–0.15 × 0.35–0.46	0.05–0.18 × 0.24–0.35	0.08–0.21 × 0.30–0.43
PRS	0.36–0.82 × 0.20–0.28	0.55–0.97 × 0.25–0.40	0.50–0.88 × 0.21–0.34	0.75–1.30 × 0.30–0.48
LS	0.51–1.01 × 0.16–0.38	0.55–1.25 × 0.15–0.42	0.62–1.25 × 0.15–0.31	0.62–1.83 × 0.10–0.40
ATS	0.45–0.79 × 0.40–0.66	–	0.49–1.15 × 0.30–0.75	–
PTS	0.49–0.89 × 0.41–0.65	–	0.53–1.00 × 0.34–0.70	–
ES	–	0.045–0.088 × 0.018–0.028	–	0.070–0.080 × 0.020–0.026
Host	*Bufo melanostictus*		*Bufo melanostictus*, *Rana guentheri*, *R. taipehensis*, *Paa verrucospinosa*, *P. mutus*	
Locality	China		Vietnam	

*TL* Length of trunk, *TW* maximum width of trunk, *PS* size of proboscis, *NHR* number of longitudinal rows of proboscis hooks, *NHPR* number of hooks per longitudinal row, *NS* neck size, *PRS* size of proboscis receptacle, *LS* size of lemnisci, *ATS* size of anterior testis, *PTS* size of posterior testis, *ES* egg size

^a^All measurements in table are in millimeters

*Host of present specimens*: *Bufo melanostictus* (Schneider) (Anura: Bufonidae).

*Locality of present specimens*: Shuiman, Wuzhishan Mountain, Hainan Province.

*Site in host*: Intestine.

*Prevalence and intensity of infections*: Ten out of 39 individuals of *B. melanostictus* infected with intensity of 1–8 (mean 2.7) acanthocephalans.

*Voucher specimens*: Nine male, 10 females (HBNU–A–A2023015ZL); deposited in the College of Life Sciences, Hebei Normal University, Hebei Province, China.

*Representative DNA sequences*: Representative genetic data were deposited in the National Center for Biotechnology Information (NCBI) database (http://www.ncbi.nlm.nih.gov) under the accession numbers: PP447459, PP447460 (18S); PP466919, PP466920 (ITS); PP476963, PP476964 (*cox1*); PP486177, PP486178 (*cox2*); PP471562, PP471563 (12S); PP476192 (mitogenome).

**Remarks**

In the present study, the morphology of *P. nguyenthileae* was observed using light microscopy and, for the first time, scanning electron microscopy based on newly collected specimens from *Bufo melanostictus* (Schneider) in China. The morphology and morphometric data of the present specimens agreed well with the original description of *P. nguyenthileae* [1], including the size of the trunk, neck, lemnisci, proboscis receptacle and testis; the size and armature of the proboscis; the number and arrangement of cement glands (4 long and 4 clavate); and the size of eggs (See Table 1 for details). Furthermore, the present specimens and some of Amin et al.’s specimens were both collected from the same host *B. melanostictus* in a neighboring region of Southeast Asia [1]. Consequently, we considered that the present specimens belong to *P. nguyenthileae*. The position of the genital pore of this species appears variable in both males and females from different hosts. The present study also sequenced the partial *cox1*, *cox2* and 12S data of *P. nguyenthileae* for the first time, which displayed low level of intraspecific variation only in the partial *cox*1 (1.52%) regions between different individuals of *P. nguyenthileae*. These genetic data are very useful for molecular identification of this species in the future. This is the first report of this species in China.

### Revised key to the species of the genus *Pseudoacanthocephalus*

1. Proboscis oval, with 20–22 longitudinal hook rows and 10–12 hooks in each longitudinal row.................................. *P. rauschi*
2. Proboscis cylindrical................................. 2
3. Proboscis distinctly elongated (0.86 mm), with 13–15 hooks in each longitudinal row............................................. *P. elongatus*
4. Proboscis not elongated, with less than 13 hooks in each longitudinal row............................................... 3
5. Proboscis with 8–12 longitudinal hook rows............. *P. xenopeltidis*
6. Proboscis with 12–22 longitudinal hook rows............. 4
7. Parasitic in chameleons and snakes........................ 5
8. Parasitic in amphibians................................ 6
9. Trunk of female 10.0–20.0 mm long, proboscis with 3 hooks each longitudinal row in male...................................... *P. rhampholeonotos*
10. Trunk of female 7.50 mm long, proboscis with 4 hooks each longitudinal row in male........................................... *P. bigueti*
11. Eggs with polar prolongations.............................. 7
12. Eggs without polar prolongations.......................... 8
13. Proboscis with 14 longitudinal hook rows.................* P. lucidus*
14. Proboscis with 14–16 longitudinal hook rows..............* P. toshimai*
15. Testes pre-equatorial................................... 9
16. Testes equatorial or post-equatorial....................... 10
17. Proboscis with 12–13 longitudinal hook rows............ *P. smalesae*
18. Proboscis with 16–18 longitudinal hook rows....... *P. previatesticulus* sp. n.
19. Trunk of female narrowed in middle region and distinctly widened towards posterior end......................................... *P. nickoli*
20. Trunk of female not narrowed in middle region and not widened towards posterior end........................................... 11
21. Proboscis hooks increase progressively in length from apical to basal position.............................................. 12
22. Proboscis hooks in middle region longer than that in apical and basal position.............................................. 13
23. Proboscis with 12–15 longitudinal hook rows............. *P. reesei*
24. Proboscis with 16–18 longitudinal hook rows........ *P. nguyenthileae*
25. Male with eight cement glands........................... 14
26. Male with six cement glands........................... 15
27. Proboscis with 13 longitudinal hook rows............. *P. coniformis*
28. Proboscis with 18–20 longitudinal hook rows........ *P. sichuanensis* sp. n.
29. Trunk of male only 2.60–3.20 mm long................ *P. perthensis*
30. Trunk of male over 3.50 mm long....................... 16
31. Lemnisci shorter or equal in length with proboscis receptacle..... 17
32. Lemnisci longer than proboscis receptacle.................. 18
33. Trunk of female about 7.0 mm long, proboscis 0.47 × 0.30 mm in size.............................................. *P. betsileo*
34. Trunk of female 11.0–13.1 mm long, proboscis 0.50–0.55 × 0.37–0.43 mm in size...........................................*P. caucasicus*
35. Cement glands pyriform................................... 19
36. Cement glands long, tubular or filiform....................... 21
37. Proboscis with 18–19 longitudinal hook rows, trunk of male 10.7–15.1 mm long..................................... *P. caspanensis*
38. Proboscis with 14–18 longitudinal hook rows, trunk of male 4.00–9.75 mm long.................................................. 20
39. Proboscis usually with 15–16 longitudinal hook rows, distributed in South America.........................................*P. lutzi*
40. Proboscis with 14 longitudinal hook rows, distributed in India.. *P. shillongensis*
41. Trunk of female only 6.50–10.7 mm long, proboscis with 6–7 hooks each longitudinal row.......................... *P. bufonincola*
42. Trunk of female 14.0–28.0 mm long, proboscis with 4–6 hooks each longitudinal row.................................................... 22
43. Proboscis usually with 14 longitudinal hook rows..............*P. goodmani*
44. Proboscis usually with 16 longitudinal hook rows............. *P. bufonis*

### Species delimitation of *Pseudoacanthocephalus* spp.

**Fig. 9:**
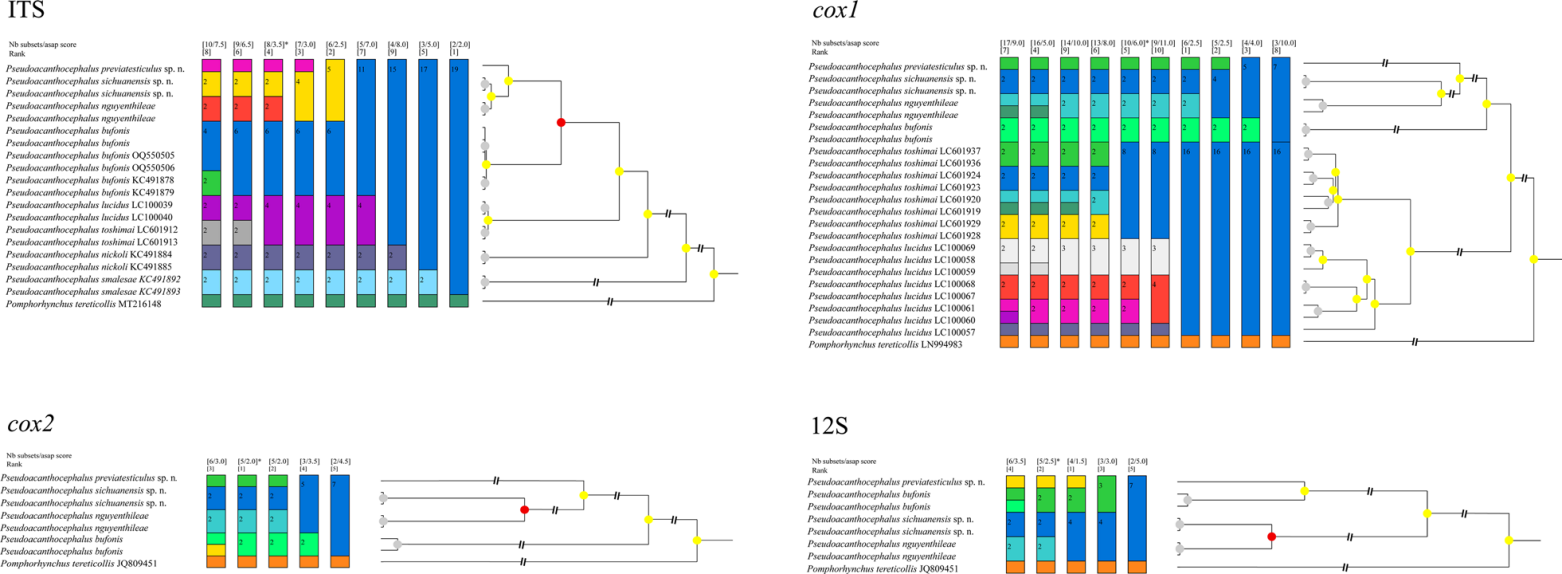
Assemble Species by Automatic Partitioning (ASAP) analyses of *Pseudoacanthocephalus* spp. based on 4 different nuclear and mitochondrial genetic markers. *Pomphorhychus tereticollis* was chosen as the outgroup. The asterisk (*) indicates the best result according the lowest score and optimal recommendation by ASAP. 12S, Small subunit ribosomal RNA sequence; *co*x*1*, *cox2*, cytochrome* c* oxidase subunit 1 and 2, respectively; ITS, internal transcribed spacer

**Fig. 10:**
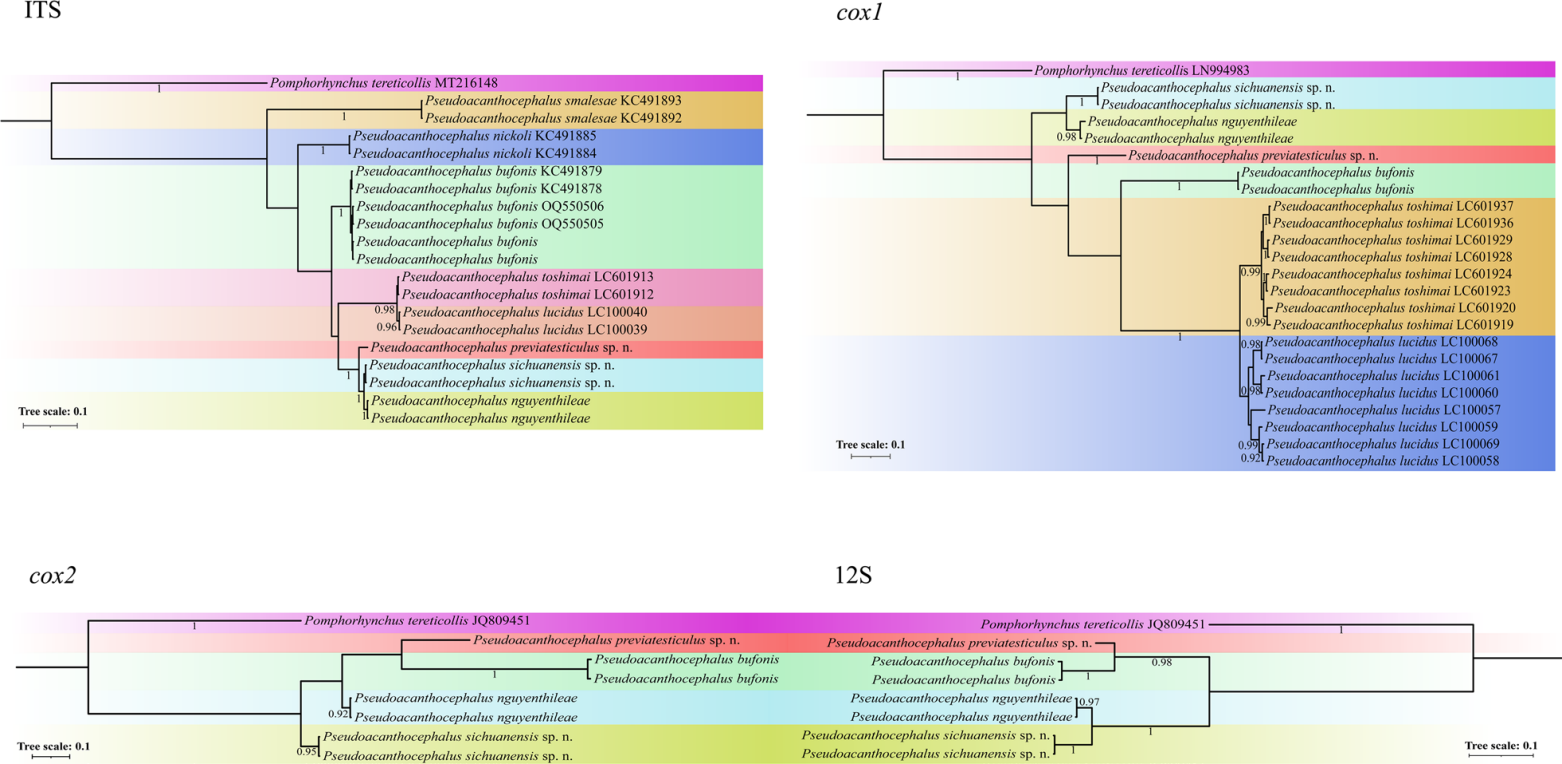
Bayesian inference of *Pseudoacanthocephalus* spp. based on 4 different nuclear and mitochondrial genetic markers (12S, small subunit ribosomal RNA sequence; *co*x*1*, *cox2*, cytochrome* c* oxidase subunit 1 and 2, respectively; ITS, internal transcribed spacer). *Pomphorhychus tereticollis* was chosen as the outgroup. Bayesian posterior probabilities values ≥ 0.70 are shown on nodes

The present results of the ASAP analyses using the *cox1*, *cox2* and 12S sequences all supported the species partition of *P. sichuanensis*, *P. previatesticulus* and *P. nguyenthileae*, but the ASAP results using ITS data did not support *P. sichuanensis* sp. n. and *P. nguyenthileae* representing two distinct species (Fig. 9). BI based on the ITS, *cox1*, *cox2* and 12S sequences all showed samples of *P. sichuanensis*, *P. previatesticulus* and *P. nguyenthileae* forming distinct clades, with *P. sichuanensis* being sister to *P. nguyenthileae* (Fig. 10).

### Mitogenomes of *Pseudoacanthocephalus sichuanensis *and* P. nguyenthileae*

**Fig. 11:**
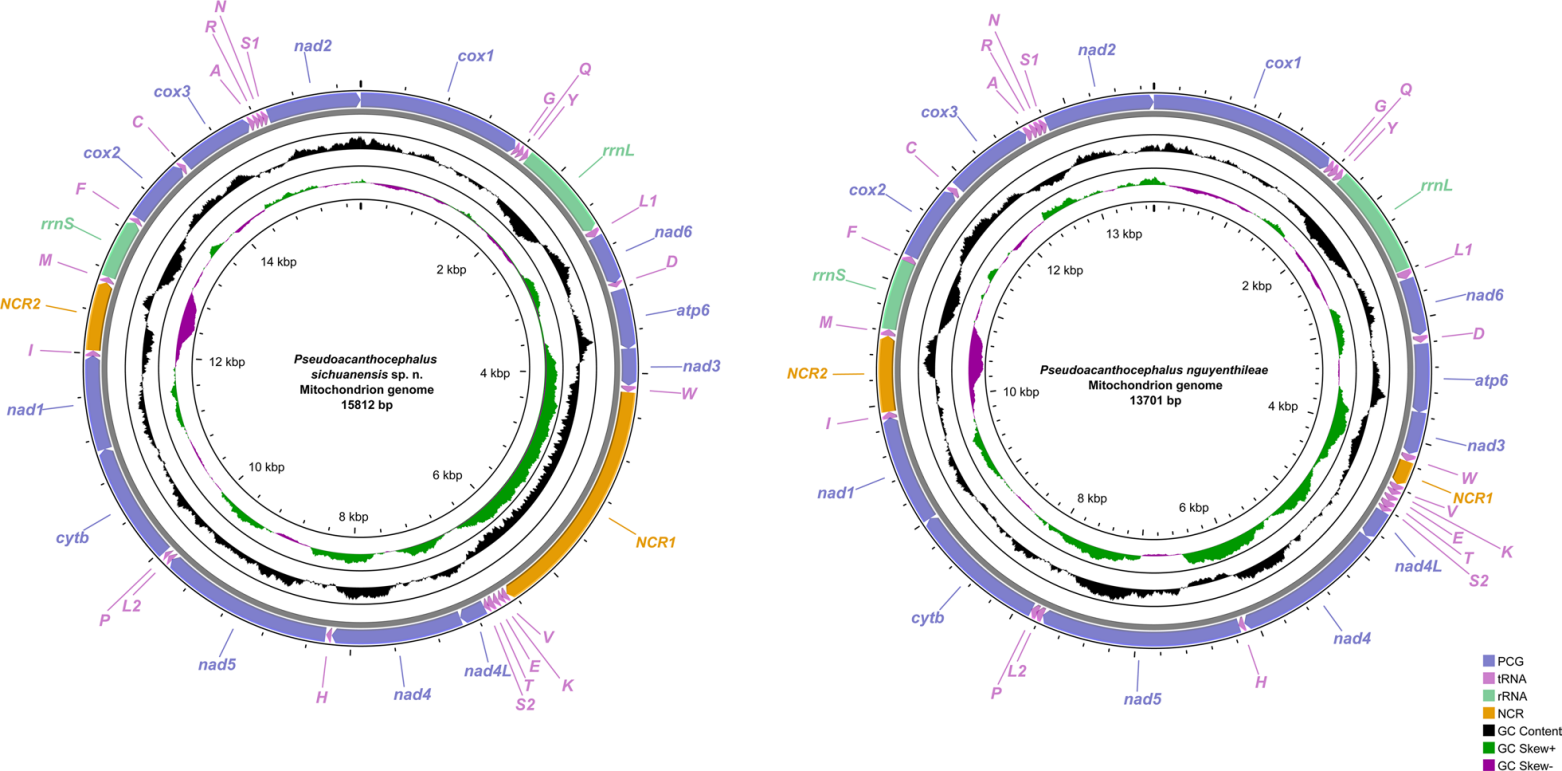
Gene map of the mitochondrial genome of *Pseudoacanthocephalus sichuanensis* sp. n. and *Pseudoacanthocephalus nguyenthileae*. NCR, Non-coding region; PCG, protein-coding gene; rRNA, ribosomal RNA; tRNA, transfer RNA

The complete mitogenomes of *P. sichuanensis* and *P. nguyenthileae* have 15812 bp and 13701 bp, respectively, both of which include 36 genes, containing 12 PCGs (*cox1–3, cytb*, *nad1–6*, *nad4L* and *atp6*, missing *atp8*), 22 transfer RNA (tRNA) genes and two rRNA genes (*rrnL* and *rrnS*) (Fig. 11; Additional file 7: Table S4; Additional file 8: Table S5). Two non-coding regions (NCRs) are present in the mitogenomes of *P. sichuanensis* and *P. nguyenthileae* (NCR1 is 2282 bp in *P. sichuanensis* vs only 202 bp in *P. nguyenthileae*, both between *tRNA-Trp* and *tRNA-Val*; NCR2 is 650 bp in *P. sichuanensis *vs 618 bp in *P. nguyenthileae*, both between *tRNA-Ile* and *tRNA-Met*) (Fig. 11; Additional file 7: Table S4; Additional file 8: Table S5). All genes are transcribed from the same strand. The overall A + T content in the mitogenomes of *P. sichuanensis* and *P. nguyenthileae* is 56.8% and 56.3%, respectively, with both displaying a strong nucleotide compositional bias toward A + T. The nucleotide content of *P. sichuanensis* and *P. nguyenthileae* mitogenomes are provided in Additional file 9: Table S6.

Taken together, the 12 PCGs of the mitogenomes of *P. sichuanensis* and *P. nguyenthileae* are 10,179 bp and 10,206 bp in length (excluding termination codons), with each gene ranging in size from 240 bp (*nad4L*) to 1627 bp (*nad5*), which encoded 3391 and 3401 amino acids, respectively (Additional file 7: Table S4; Additional file 8: Table S5). Among the 12 PCGs of *P. sichuanensis* and *P. nguyenthileae*, six genes (*cox1*, *cox3*, *nad4L*, *nad1*, *nad2* and *nad5*) used GTG as the start codon, whereas two genes (*cox2* and *atp6*) in *P. sichuanensis* and four genes (*cox2*, *nad3*, *nad6* and *atp6*) in *P. nguyenthileae* used ATG as the start codon. TTG was used by the *nad4* and *cytb* genes in both species. TTG was used by the *nad6* gene and ATA was used by the *nad3* as the start codon in *P. sichuanensis*. TAA was the most commonly used termination codon in both species (4 genes [*cox3*, *nad2*, *nad3*, *nad4L*] in *P. sichuanensis * vs 3 genes [*cox3*, *nad2*, *nad3*] in *P. nguyenthileae*); two genes (*cox1* and *cytb*) in *P. sichuanensis* and three genes (*cox1*, *nad4L* and *cytb*) in *P. nguyenthileae* used TAG as termination codon. The incomplete termination codon T was inferred for the *cox2*, *nad1*, *nad4*, *nad5*, *nad6* and *atp6* genes in both species. Data on the component and usages of codons in the mitogenomes of *P. sichuanensis* and *P. nguyenthileae* are provided in Additional file 1: Figure S1; Additional file 7: Table S4; Additional file 8: Table S5.

There are 22 tRNAs and two rRNAs (*rrnL* located between *tRNA-Tyr* and *tRNA-Leu1*; *rrnS* located between *tRNA-Met* and *tRNA-Phe*) in the mitogenomes of *P. sichuanensis* and *P. nguyenthileae* (Fig. 11; Additional file 7: Table S4; Additional file 8: Table S5). The length of the 22 tRNAs and their anticodon secondary structures of *P. sichuanensis* and *P. nguyenthileae* are provided in Additional file 2: Figure S2; Additional file 3: Figure S3; Additional file 7: Table S4; Additional file 8: Table S5.

The gene arrangement of the 36 genes in the mitogenomes of *P. sichuanensis* and *P. nguyenthileae* are identical, both in the following order: *cox1*, *tRNA-Gly*, *tRNA-Gln*, *tRNA- Tyr*, *rrnL*, *tRNA-Leu1*, *nad6*, *tRNA-Asp*, *atp6*, *nad3, tRNA-Trp*, *tRNA-Val*, *tRNA-Lys*, *tRNA-Glu*, *tRNA-Thr*, *tRNA-Ser2*, *nad4L*, *nad4*, *tRNA-His*, *nad5*, *tRNA-Leu2*, *tRNA-Pro*, *cyt*b, *nad1*, *tRNA-Ile*, *tRNA-Met*, *rrnS*, *tRNA-Phe*, *cox2*, *tRNA-Cys*, *cox3*, *tRNA-Ala*, *tRNA-Arg*, *tRNA-Asn*, *tRNA-Ser1*, *nad2* (Fig. 11).

### Mitogenomic phylogenetic analyses

**Fig. 12:**
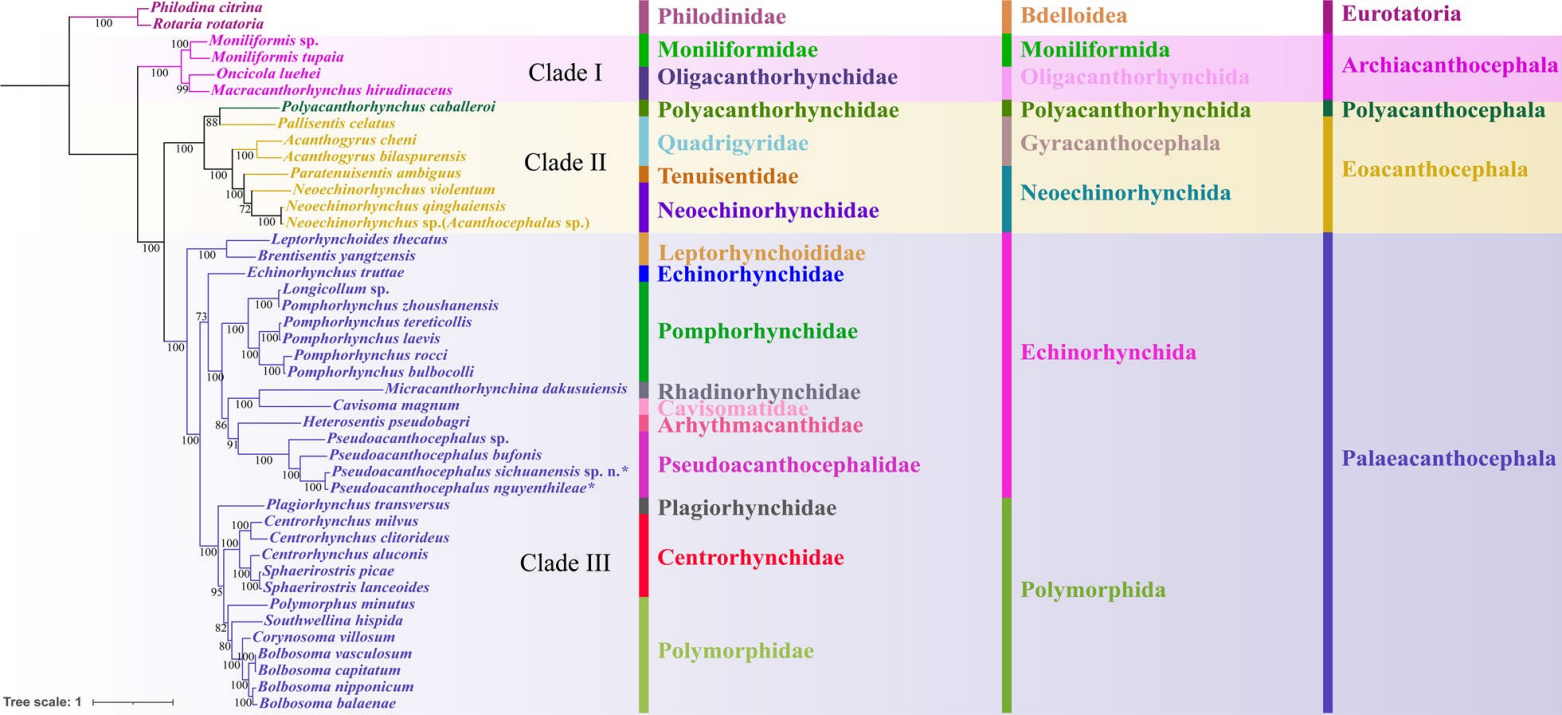
Phylogenetic relationships among acanthocephalans constructed using maximum likelihood inference based on the amino acid sequences of 12 protein-coding genes of mitochondrial genomes. *Rotaria rotatoria* (Bdelloidea: Philodinidae) and *Philodina citrina* (Bdelloidea: Philodinidae) were chosen as the outgroup. Bootstrap values ≥ 70 are shown in the phylogenetic trees

**Fig. 13:**
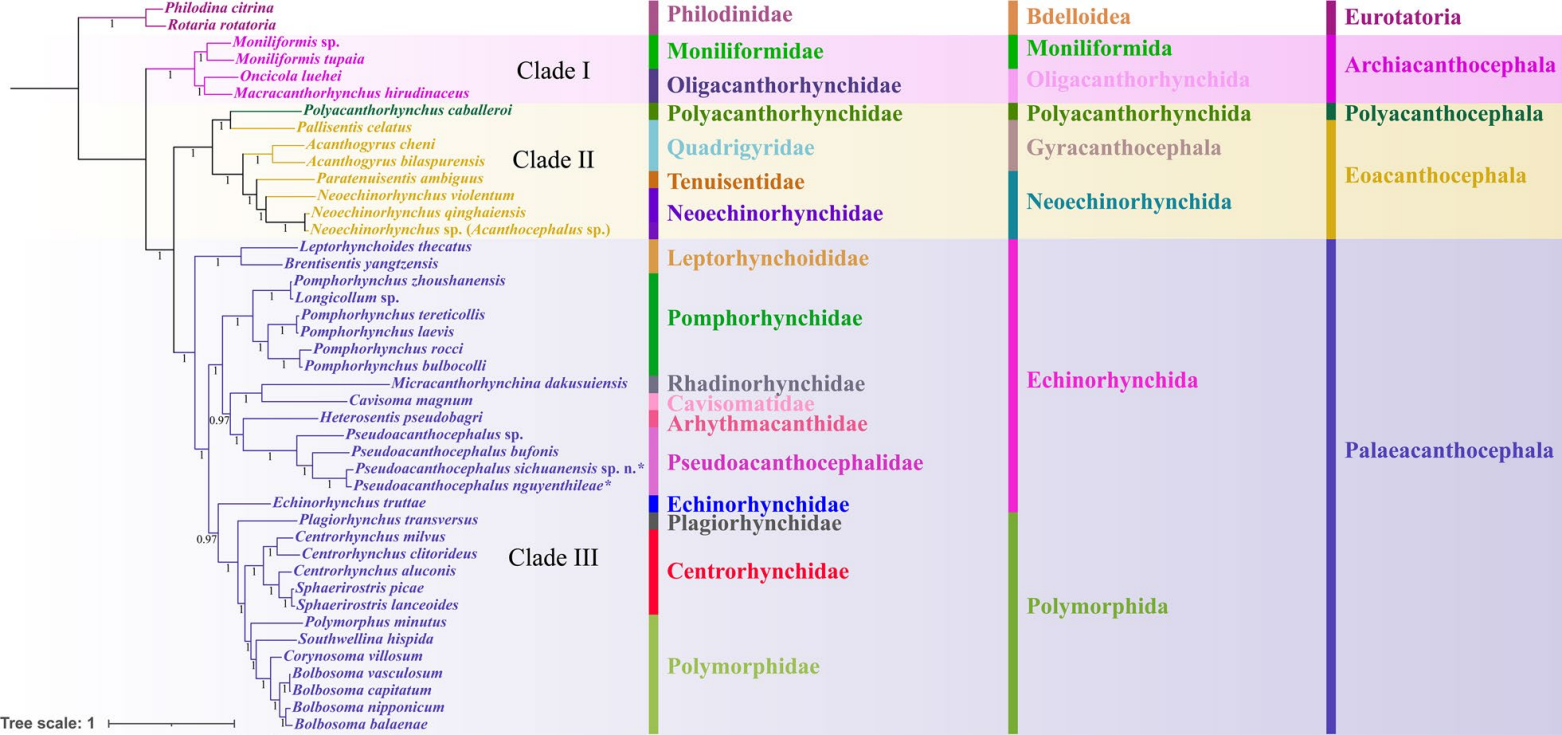
Phylogenetic relationships among acanthocephalans constructed using Bayesian inference based on the amino acid sequences of 12 protein-coding genes of mitochondrial genomes. *Rotaria rotatoria* (Bdelloidea: Philodinidae) and *Philodina citrina* (Bdelloidea: Philodinidae) were chosen as the outgroup. Bayesian posterior probabilities values ≥ 0.90 are shown in the phylogenetic trees

The topologies of phylogenetic trees using ML and BI methods were found to be nearly identical (Figs. 12, 13), which showed that the representatives of Acanthocephala formed into three large monophyletic clades, representing the classes Archiacanthocephala (Clade I), Eoacanthocephala (Clade II) and Palaeacanthocephala (Clade III), respectively. Among these, the Archiacanthocephala (including *Macracanthorhynchus hirudinaceus*, *Moniliformis* spp. and *Oncicola luehei*) was the basal clade. The Polyacanthocephala (including only *Polyacanthorhynchus caballeroi*) nested with the representatives of the Eoacanthocephala, and formed a sister lineage to *Pallisentis celatus* (Gyracanthocephala: Quadrigyridae). The phylogenetic results also showed that the family Quadrigyridae (Gyracanthocephala) in Clade II, and the order Echinorhynchida in Clade III are non-monophyletic. In Clade III, the Polymorphida is monophyletic. The family Pseudoacanthocephalidae (including *Pseudoacanthocephalus bufonis*, *P. nguyenthileae*, *Pseudoacanthocephalus* sp. and *P. sichuanensis*) was sister to the representative of the family Arhythmacanthidae (including *Heterosentis pseudobagri*). In the genus *Pseudoacanthocephalus*, *P. sichuanensis* is closer to *P. nguyenthileae* than *Pseudoacanthocephalus* sp. and *P. bufonis* (Figs. 12, 13).

## Discussion

The ASAP is a new powerful and efficient method for species delimitation that has been widely used for the integrative taxonomy of various taxa [36–41]. The optimal results of ASAP analyses using the *cox1*, *cox2* and 12S sequences supported *P. sichuanensis* and *P. previatesticulus* representing distinct species from the other *Pseudoacanthocephalus* species. However, the optimal ASAP results using ITS data did not support the species partition of *P. sichuanensis* and *P. nguyenthileae*, together with *P. lucidus* and *P. toshimai*. The results of the present study can be easily understood when we consider the presence of only 0.17–0.65% nucleotide divergence in ITS region between *P. sichuanensis* and *P. nguyenthileae*, and 0.83% nucleotide divergence between *P. lucidus* and *P. toshimai*, which is distinctly lower than the level of nucleotide divergence in *cox1*, *cox2* and 12S sequences. The results of our BI analyses based on the ITS, *cox1*, *cox2* and 12S sequences all supported *P. sichuanensis* and *P. previatesticulus* representing distinct genetic lineages from the other *Pseudoacanthocephalus* species. Although the 18S and 28S sequences with slow evolutionary rate are unsuitable for species delimitation of *Pseudoacanthocephalus* species, especially the cryptic species or sibling species, we also provided the 18S and 28S data of *P. sichuanensis*, *P. previatesticulus* and *P. nguyenthileae* for the first time, which will be very useful and valuable reference for investigating the phylogenetic relationships of higher taxa within the Acanthocephala in the future.

The mitogenomes of acanthocephalans are very useful for investigation molecular phylogeny, population genetics and evolutionary history, but the number of sequenced mitogenomes of acanthocephalans is still very limited. In the family Pseudoacanthocephalidae, the complete mitogenomes are only known for *P. bufonis* and *Pseudoacanthocephalus* sp. [12]. The size of the complete mitogenome of *P. sichuanensis* (15,812 bp) is distinctly larger than that of other acanthocephalans, with the exception of that of *Micracanthorhynchina dakusuiensis* (16,309 bp) and *Centrorhynchus clitorideus* (15,884 bp) [42, 43]. The composition of the mitogenomes of *P. sichuanensis* and *P. nguyenthileae* were reported to be typical of most acanthocephalan mitogenomes, except for *Leptorhynchoides thecatus*, which has the *atp8* gene [44]. The gene arrangement in the mitogenomes of *P. sichuanensis* and *P. nguyenthileae* is the same as that of *Pseudoacanthocephalus* sp., *P. bufonis*, *Moniliformis* sp., *Acanthogyrus cheni*, *Brentisentis yangtzensis* and *Polymorphus minutus* [12, 45–48]. The NCR1 (2282 bp) in the mitogenome of *P. sichuanensis* is distinctly longer than that of other acanthocephalans, with the exception of that of *C. clitorideus* [43]*.*

The order Echinorhynchida is a large group of acanthocephalans and currently includes approximately 15 families [9, 10, 49]. However, the phylogenetic relationships of these families in Echinorhynchida remain unsolved due to the scarcity and inaccessibility of genetic data for some taxa. The present mitogenomic phylogeny revealed that the Echinorhynchida is non-monophyletic, which is consistent with some previous studies [11, 12, 43, 45, 50–54]. The authors of a previous study suggested the resurrection of the family Pseudoacanthocephalidae [10], which was supported by some recent molecular phylogenetic results [11, 12]. The present mitogenomic phylogeny including two additional species of Pseudoacanthocephalidae further confirmed the validity of the family Pseudoacanthocephalidae, and indicated that Pseudoacanthocephalidae is sister to the family Arhythmacanthidae, which agreed well with the phylogenetic results [11, 12]. It is surprising that *Acanthocephalus* sp. (MT345686) belonging to the recently resurrected family Paracanthocephalidae clustered together with the family Neoechinorhynchidae of the class Eoacanthocephala in the present phylogeny. We speculated the voucher specimen of *Acanthocephalus* sp. (MT345686, unpublished data) was probably identified erroneously (genetic evidence of *cox1* and *cox2* indicated *Acanthocephalus* sp. belonging to the genus *Neoechinorhynchus*).

## Conclusions

Two new species of the genus *Pseudoacanthocephalus*, namely *P. sichuanensis* sp. n. and *P. previatesticulus* sp. n., were identified based on integrated evidence. This is the first report of *Pseudoacanthocephalus nguyenthileae* in China. A revised key to the species of the genus *Pseudoacanthocephalus* was provided. Molecular analyses indicated that the mitochondrial *cox1*, *cox2* and 12S genes as genetic markers seem to be more suitable for species delimitation of the genus *Pseudoacanthocephalus* than the nuclear ITS region. BI results suggested a close affinity between *P. sichuanensis* and *P. nguyenthileae*. The characterization of the complete mitochondrial genomes of *P. sichuanensis* and *P. nguyenthileae* were reported for the first time. Mitogenomic phylogenetic results further confirmed the validity of the family Pseudoacanthocephalidae.

## Supplementary Information

**Additional file 1: Figure S1.** Relative synonymous codon usage of *P. sichuanensis* sp. n. and *P. nguyenthileae*. Codon families (in alphabetical order) are provided below the horizontal axis. Values on the top of each bar represent amino acid usage in percentage.

**Additional file 2: Figure S2. **Inferred secondary structures of 22 tRNAs in the mitogenome of *P. sichuanensis* sp. n. (Watson–Crick bonds indicated by lines, GU bonds indicated by dots, red bases representing anticodons).

**Additional file 3: Figure S3. **Inferred secondary structures of 22 tRNAs of in the mitogenome of *P. nguyenthileae* (Watson–Crick bonds indicated by lines, GU bonds indicated by dots, red bases representing anticodons).

**Additional file 4: Table S1.** Primers and cycling conditions used for amplification of target regions of *Pseudoacanthocephalus* species.

**Additional file 5: Table S2.** Detailed information of the representatives of Acanthocephala included in the present phylogeny.

**Additional file 6: Table S3.** The partitioning schemes and the optimal amino acid substitution model selected for each combination of partition for the BI and ML inference.

**Additional file 7: Table S4.** Annotations and gene organization of *P. sichuanensis* sp. n. Positive number in the “Gap or overlap” column indicates the length of intergenic sequence, and the negative number indicates the length (absolute number) that adjacent genes overlap (negative sign).

**Additional file 8: Table S5.** Annotations and gene organization of *P. nguyenthileae*. Positive number in the “Gap or overlap” column indicates the length of intergenic sequence, and the negative number indicates the length (absolute number) that adjacent genes overlap (negative sign).

**Additional file 9: Table S6.** Base composition and skewness of *Pseudoacanthocephalus sichuanensis* sp. n. and *P. nguyenthileae*.

## Data Availability

No datasets were generated or analysed during the current study.

## Abbreviations

12S: Small subunit ribosomal RNA sequence
28S: Large ribosomal subunit
ASAP: Assemble Species by Automatic Partitioning
BI: Bayesian inference
BIC: Bayesian information criterion
BPP: Bayesian posterior probabilities
BS: Bootstrap support
CAI: Codon adaptation index
*cox1*: Cytochrome* c* oxidase subunit 1
cox2: Cytochrome* c* oxidase subunit 2
ITS: Internal transcribed spacer
ML: Maximum likelihood
PCG: Protein-coding gene
