# Supplementary figures and images for: Integrative taxonomy of the genus Pseudoacanthocephalus (Acanthocephala: Echinorhynchida) in China, with the description of two new species and the characterization of the mitochondrial genomes of Pseudoacanthocephalus sichuanensis sp. n. and Pseudoacanthocephalus nguyenthileae
Source: Parasit Vectors. 2024 Dec 27;17:541. doi: 10.1186/s13071-024-06528-7 (PMC11681651; doi:10.1186/s13071-024-06528-7)

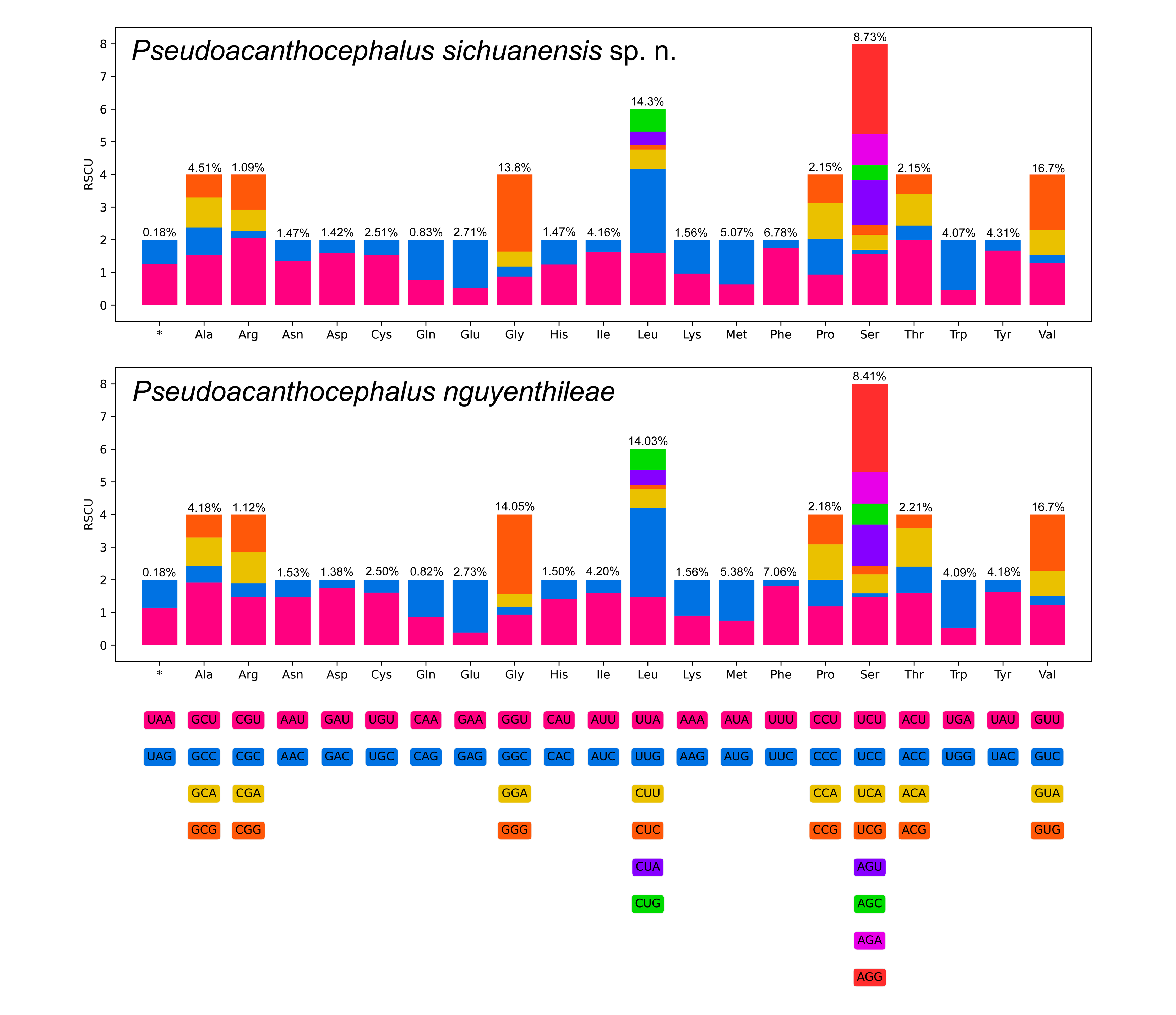

Supplement: Supplementary file 1 — Additional file 1: Figure S1. Relative synonymous codon usage of P. sichuanensis sp. n. and P. nguyenthileae. Codon families (in alphabetical order) are provided below the horizontal axis. Values on the top of each bar represent amino acid usage in percentage. [file 13071_2024_6528_MOESM1_ESM.tif]

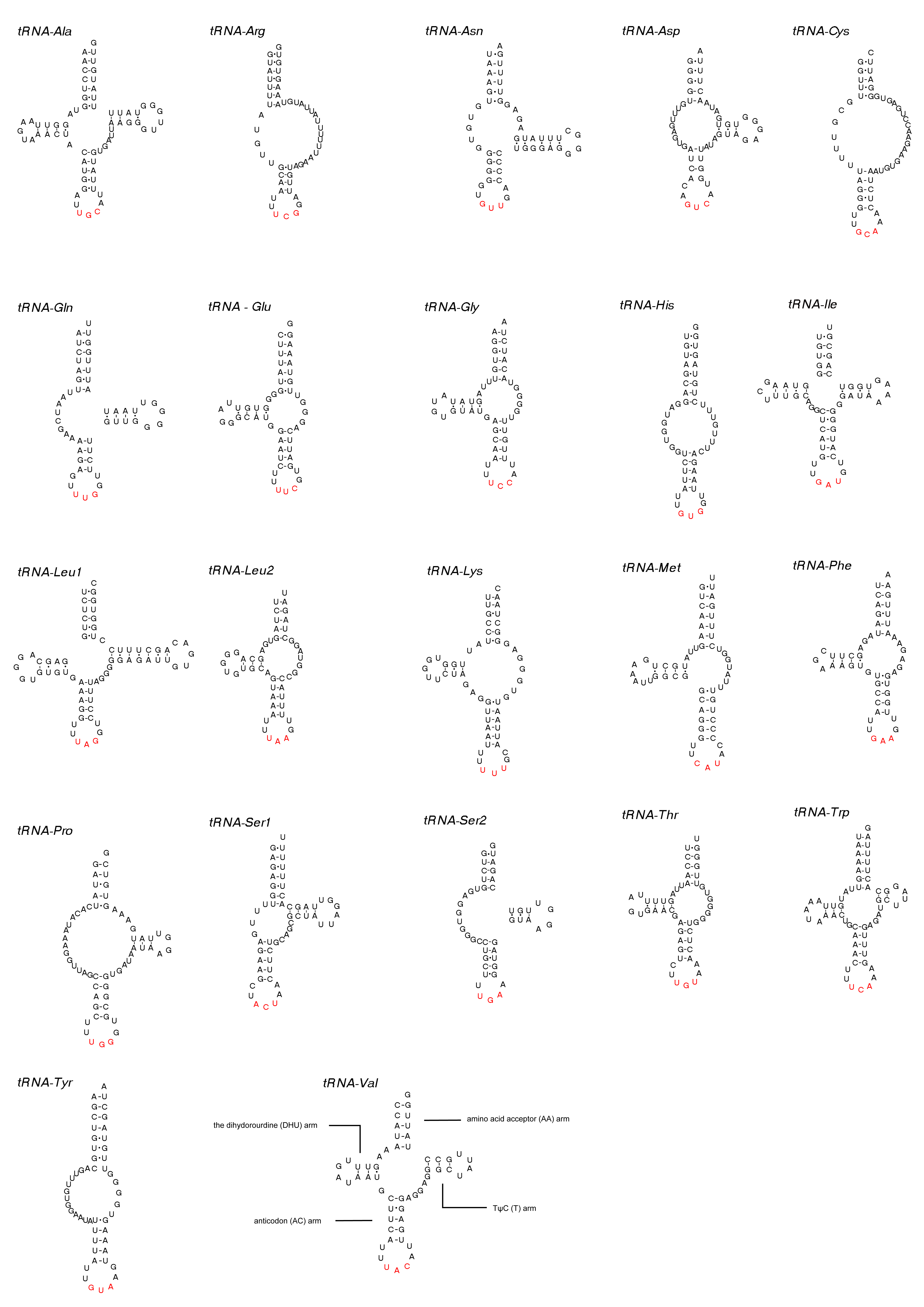

Supplement: Supplementary file 2 — Additional file 2: Figure S2. Inferred secondary structures of 22 tRNAs in the mitogenome of P. sichuanensis sp. n. (Watson–Crick bonds indicated by lines, GU bonds indicated by dots, red bases representing anticodons). [file 13071_2024_6528_MOESM2_ESM.tif]

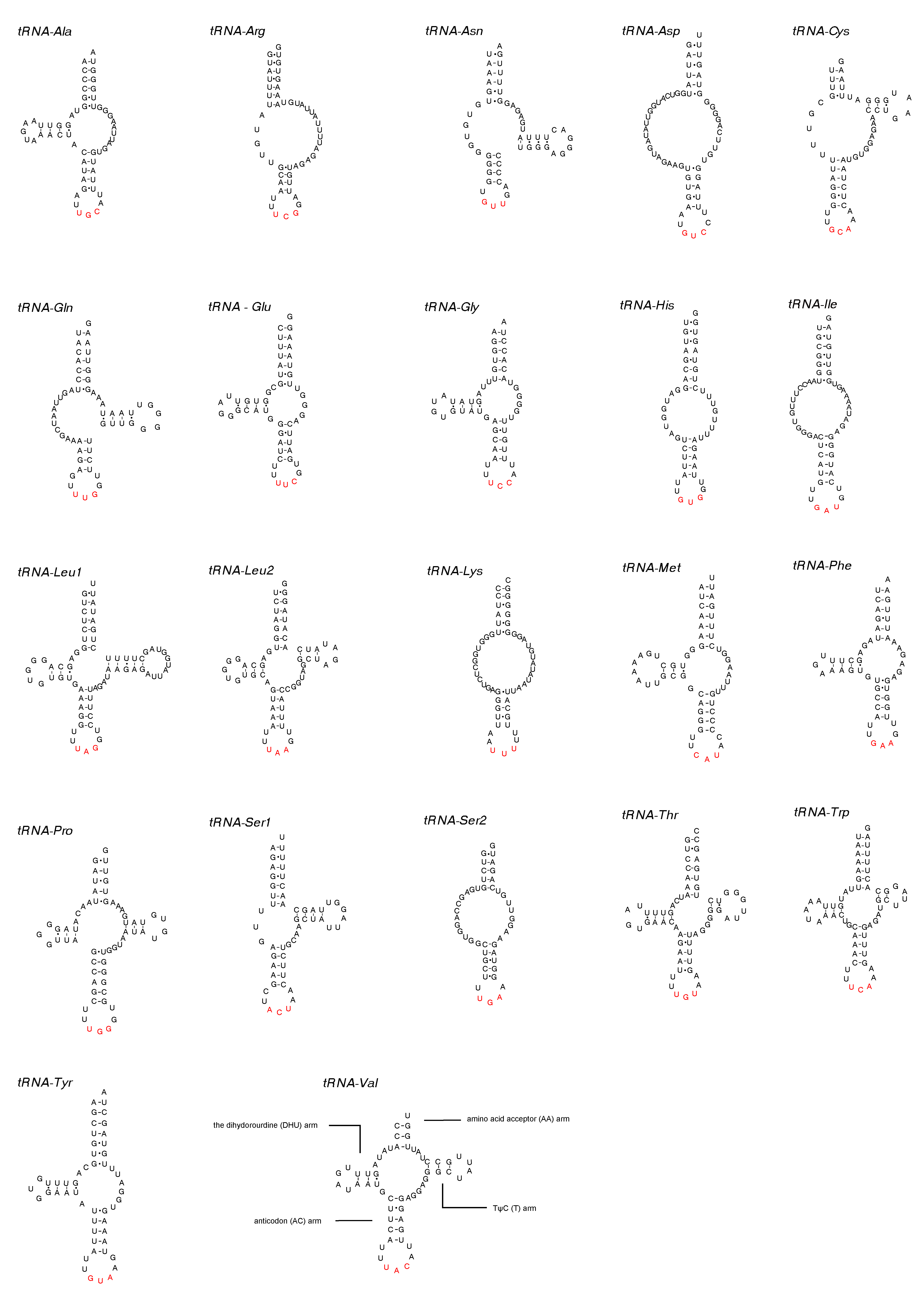

Supplement: Supplementary file 3 — Additional file 3: Figure S3. Inferred secondary structures of 22 tRNAs of in the mitogenome of P. nguyenthileae (Watson–Crick bonds indicated by lines, GU bonds indicated by dots, red bases representing anticodons). [file 13071_2024_6528_MOESM3_ESM.tif]
