# Supplementary material for: Integrative taxonomy of the genus Pseudoacanthocephalus (Acanthocephala: Echinorhynchida) in China, with the description of two new species and the characterization of the mitochondrial genomes of Pseudoacanthocephalus sichuanensis sp. n. and Pseudoacanthocephalus nguyenthileae
Source: Parasit Vectors. 2024 Dec 27;17:541. doi: 10.1186/s13071-024-06528-7 (PMC11681651; doi:10.1186/s13071-024-06528-7)
Supplement: Supplementary file 4 — Additional file 4: Table S1. Primers and cycling conditions used for amplification of target regions of Pseudoacanthocephalus species. [file 13071_2024_6528_MOESM4_ESM.docx]

**Supplementary Table S1.** Primers and cycling conditions used for amplification of target regions of *Pseudoacanthocephalus* species.

| Primers | Primer sequences (5ʹ-3ʹ) | Target region | Cycling condition | Reference |
| --- | --- | --- | --- | --- |
| 18S-F1 forward | 5′-AGATTAAGCCATGCATGCGTAAG-3′ | 18S | 94°C for 2min  94°C for 20s  60°C for 20s  65°C for 50s (25 cycles)  65°C for 5min | [1] |
| 18S-R1 reverse | 5′-GAATTACCGCGGCTGCTGG-3′ |  |  |  |
| 18S-F2 forward | 5′-GCCGCGGTAATTCCAGCTC-3′ |  |  |  |
| 18S-R2 reverse | 5′-CTGGTGTGCCCCTCCGTC-3′ |  |  |  |
| 18S-F3 forward | 5′-CGGGGGGAGTATGGTTGC-3′ |  |  |  |
| 18S-R3 reverse | 5′-TGATCCTTCTGCAGGTTCACCTAC-3′ |  |  |  |
| 28S-F1 forward | 5′-CAAGTAACCGTGAGGGAAAGTTGC-3′ | 28S | 94°C for 3min  94°C for 1min  55°C for 1min  72°C for 1min (35 cycles)  72°C for 7min | [2] |
| 28S-R1 reverse | 5′-CAGCTATCCTGAGGGAAAC-3′ |  |  |  |
| 28S-F2 forward | 5′-ACCCGAAAGATGGTGAACTATG-3′ |  |  |  |
| 28S-R2 reverse | 5′-CTTCTCCAAC(T/G)TCAGTCTTCAA-3′ |  |  |  |
| 28S-F3 forward | 5′-CTAAGGAGTGTGTAACAACTCACC-3′ |  |  |  |
| 28S-R3 reverse | 5′-AATGACGAGGCATTTGGCTACCTT-3′ |  |  |  |
| 28S-F4 forward | 5′-GATCCGTAACTTCGGGAAAAGGAT-3′ |  |  |  |
| 28S-R4 reverse | 5′-CTTCGCAATGATAGGAAGAGCC-3′ |  |  |  |
| ITS-F forward | 5′-GTCGTAACAAGGTTTCCGTA-3′ | ITS | 94°C for 2min  94°C for 20s  51°C for 20s  65°C for 50s (40 cycles)  65°C for 5min | [3] |
| ITS-R reverse | 5′-TATGCTTAAATTCAGCGGGT-3′ |  |  |  |
| *cox1*-F forward | 5′-AGTTCTAATCATAA(R)GATAT(Y)GG-3′ | *cox*1 | 94°C for 5min  94°C for 1min  40°C for 1min  72°C for 1min (35 cycles)  72°C for 5min | [4] |
| *cox1*-R reverse | 5′-TAAACTTCAGGGTGACCAAAAAATCA-3′ |  |  |  |
| *cox2*-F forward | 5′-AGTAGTAGAGTCAATTTGGA-3′ | *cox*2 | 94°C for 2min  94°C for 30s  47°C for 30s  72°C for 30s (33 cycles)  72°C for 2min | Present study |
| *cox2*-R reverse | 5′-CTAAAACAATCGGCATAAAC-3′ |  |  |  |
| 12S-F forward | 5′-TTTTATGGGAGTAATGCTAA-3′ | 12S | 94°C for 2min  94°C for 30s  48°C for 30s  72°C for 30s (33 cycles)  72°C for 2min | Present study |
| 12S-R reverse | 5′-AGTAGAATCACTATGTTACG-3′ |  |  |  |
| 12Spng-F forward | 5′-GTTTTTCCCTATGGGAGTAG-3′ |  |  |  |
| 12Spng-R reverse | 5′-CTTTCGAAGCTCTATCTGA-3′ |  |  |  |

[1] Garey JR, Near TJ, Nonnemacher MR, et al. Molecular evidence for Acanthocephala as a subtaxon of Rotifera. J Mol Evol. 1996;43:287–92.

[2] García-Varela M, Nadler S. Phylogenetic relationships of Palaeacanthocephala (Acanthocephala) inferred from SSU and LSU rDNA gene sequences. J Parasitol. 2005;91:1401–9.

[3] Král'ová-Hromadová I, Tietz DF, Shinn AP, et al. ITS rDNA sequences of *Pomphorhynchus laevis* (Zoega in Müller, 1776) and *P. lucyi* Williams & Rogers, 1984 (Acanthocephala: Palaeacanthocephala). Syst Parasitol. 2003;56:141–5.

[4] Gómez A, Serra M, Carvalho GR, et al. Speciation in ancient cryptic species complexes: evidence from the molecular phylogeny of *Brachionus plicatilis* (Rotifera). Evolution 2002;56:1431–44.
