# Supplementary material for: Integrative taxonomy of the genus Pseudoacanthocephalus (Acanthocephala: Echinorhynchida) in China, with the description of two new species and the characterization of the mitochondrial genomes of Pseudoacanthocephalus sichuanensis sp. n. and Pseudoacanthocephalus nguyenthileae
Source: Parasit Vectors. 2024 Dec 27;17:541. doi: 10.1186/s13071-024-06528-7 (PMC11681651; doi:10.1186/s13071-024-06528-7)
Supplement: Supplementary file 5 — Additional file 5: Table S2. Detailed information of the representatives of Acanthocephala included in the present phylogeny. [file 13071_2024_6528_MOESM5_ESM.docx]

**Table S2.** Detailed information of the representatives of Acanthocephala included in the present phylogeny.

| Phylum/Class | Order | Family | Species | Accession | Length | AT% | References |
| --- | --- | --- | --- | --- | --- | --- | --- |
| **Out-group** |  |  |  |  |  |  |  |
| **Rotifera** |  |  |  |  |  |  |  |
| **Eurotatoria** | Bdelloidea | Philodinidae | *Rotaria rotatoria* | NC_013568 | 15,319 | 73.2 | [1] |
|  |  |  | *Philodina citrina* | FR856884 | 14,003 | 77.7 | [2] |
| **In-group** |  |  |  |  |  |  |  |
| **Acanthocephala** |  |  |  |  |  |  |  |
| **Archiacanthocephala** | Moniliformida | Moniliformidae | *Moniliformis* sp. | OK415026 | 14,066 | 66.2 | [3] |
|  |  |  | *Moniliformis tupaia* | OP413683 | 14,150 | 63.7 | unpublished |
|  | Oligacanthorhynchida | Oligacanthorhynchidae | *Macracanthorhynchus hirudinaceus* | NC_019808 | 14,282 | 65.2 | [2] |
|  |  |  | *Oncicola luehei* | NC_016754 | 14,281 | 60.2 | [4] |
| **Eoacanthocephala** | Gyracanthocephala | Quadrigyridae | *Acanthogyrus bilaspurensis* | MT476589 | 13,360 | 59.3 | unpublished |
|  |  |  | *Acanthogyrus cheni* | KX108947 | 13,695 | 65.3 | [5] |
|  |  |  | *Pallisentis celatus* | NC_022921 | 13,855 | 61.5 | [6] |
|  | Neoechinorhynchida | Neoechinorhynchidae | *Neoechinorhynchus violentum* | KC415004 | 13,393 | 59.4 | [7] |
|  |  |  | *Neoechinorhynchus qinghaiensis* | MW851291 | 13,271 | 65.8 | unpublished |
|  |  |  | *Neoechinorhynchus* sp. (*Acanthocephalus* sp.) | MT345686 | 13,269 | 65.1 | unpublished |
|  |  | Tenuisentidae | *Paratenuisentis ambiguus* | NC_019807 | 13,574 | 66.9 | [2] |
| **Palaeacanthocephala** | Echinorhynchida | Arhythmacanthidae | *Heterosentis pseudobagri* | OP278658 | 13,742 | 62.5 | [8] |
|  |  | Cavisomidae | *Cavisoma magnum* | MN562586 | 13,594 | 63.0 | [9] |
|  |  | Echinorhynchidae | *Echinorhynchus truttae* | NC_019805 | 13,659 | 63.1 | [2] |
|  |  | Leptorhynchoididae | *Brentisentis yangtzensis* | MK651258 | 13,864 | 68.3 | [10] |
|  |  | Pomphorhynchidae | *Pomphorhynchus bulbocolli* | JQ824371 | 13,915 | 59.9 | unpublished |
|  |  |  | *Pomphorhynchus laevis* | JQ809446 | 13,889 | 57.1 | unpublished |
|  |  |  | *Pomphorhynchus rocci* | JQ824373 | 13,845 | 60.7 | unpublished |
|  |  |  | *Pomphorhynchus tereticollis* | JQ809451 | 13,965 | 56.9 | unpublished |
|  |  |  | *Pomphorhynchus zhoushanensis* | MN602447 | 14,565 | 56.0 | unpublished |
|  |  |  | *Longicollum* sp. | OR215045 | 14,632 | 55.8 | unpublished |
|  |  | Pseudoacanthocephalidae | *Pseudoacanthocephalus bufonis* | MZ958236 | 14,056 | 58.4 | [11] |
|  |  |  | ***Pseudoacanthocephalus nguyenthileae*** | **PP476192** | 13,701 | 56.3 | present study |
|  |  |  | ***Pseudoacanthocephalus sichuanensis*** | **PP476191** | 15,812 | 56.8 | present study |
|  |  |  | *Pseudoacanthocephalus* sp. | OQ588705 | 14,883 | 61.5 | unpublished |
|  |  | Rhadinorhynchidae | *Leptorhynchoides thecatus* | NC_006892 | 13,888 | 71.4 | [12] |
|  |  |  | *Micracanthorhynchina dakusuiensis* | OP131911 | 16,309 | 56.8 | [13] |
|  | Polymorphida | Centrorhynchidae | *Centrorhynchus clitorideus* | MT113355 | 15,884 | 55.5 | [14] |
|  |  |  | *Centrorhynchus milvus* | MK922344 | 14,314 | 54.5 | [15] |
|  |  |  | *Centrorhynchus aluconis* | KT592357 | 15,144 | 54.5 | [16] |
|  |  |  | *Sphaerirostris lanceoides* | MT476588 | 13,478 | 58.0 | [17] |
|  |  |  | *Sphaerirostris picae* | MK471355 | 15,170 | 58.1 | [18] |
|  |  | Polymorphidae | *Polymorphus minutus* | MN646175 | 14,149 | 64.4 | [19] |
|  |  |  | *Southwellina hispida* | NC_026516 | 14,742 | 63.9 | [20] |
|  |  |  | *Bolbosoma nipponicum* | OR468096 | 14,296 | 60.9 | [21] |
|  |  |  | *Bolbosoma balaenae* | MZ357084 | 14,301 | 62.6 | [22] |
|  |  |  | *Bolbosoma capitatum* | MZ357085 | 14,319 | 63.9 | [22] |
|  |  |  | *Bolbosoma vasculosum* | MZ357087 | 14,313 | 63.9 | [22] |
|  |  |  | *Corynosoma villosum* | OR468095 | 14,241 | 61.0 | [21] |
|  |  | Plagiorhynchidae | *Plagiorhynchus transversus* | NC_029767 | 15,477 | 61.1 | [16] |
| **Polyacanthocephala** | Polyacanthorhynchida | Polyacanthorhynchidae | *Polyacanthorhynchus caballeroi* | NC_029766 | 13,956 | 56.3 | [16] |

[1] Min GS, Park JK. Eurotatorian paraphyly: Revisiting phylogenetic relationships based on the complete mitochondrial genome sequence of *Rotaria rotatoria* (Bdelloidea: Rotifera: Syndermata). BMC Genomics. 2009;10:e533.

[2] Weber M, Wey-Fabrizius AR, Podsiadlowski L, et al. Phylogenetic analyses of endoparasitic Acanthocephala based on mitochondrial genomes suggest secondary loss of sensory organs. Mol Phylogenet. 2013;66:182–9.

[3] Dai GD, Yan HB, Li L, *et al*. Molecular characterization of a new *Moniliformis* sp. from a plateau zokor (*Eospalax fontanierii baileyi*) in China. Front Microbiol. 2022;13:e806882.

[4] Gazi M, Sultana T, Min GS, et al. The complete mitochondrial genome sequence of *Oncicola luehei* (Acanthocephala: Archiacanthocephala) and its phylogenetic position within Syndermata. Parasitol Int. 2012;61:307–16.

[5] Song R, Zhang D, Deng S, et al. The complete mitochondrial genome of *Acanthosentis cheni* (Acanthocephala: Quadrigyridae). Mitochondrial DNA B 2016;1:797–8.

[6] Pan TS, Nie P. The complete mitochondrial genome of *Pallisentis celatus* (Acanthocephala) with phylogenetic analysis of acanthocephalans and rotifers. Folia Parasit. 2013;60:181–91.

[7] Pan T, Jiang H. The complete mitochondrial genome of *Hebesoma violentum* (Acanthocephala). Mitochondrial DNA B. 2018;3:582–3.

[8] Gao JW, Yuan XP, Jakovlic I, *et al*. The mitochondrial genome of *Heterosentis pseudobagri* (Wang & Zhang, 1987) Pichelin & Cribb, 1999 reveals novel aspects of tRNA genes evolution in Acanthocephala. BMC Genomics 2023;24:e95.

[9] Muhammad N, Li L, Suleman, et al. Characterization of the complete mitochondrial genome of *Cavisoma magnum* (Acanthocephala: Palaeacanthocephala), first representative of the family Cavisomidae, and its phylogenetic implications. Infect Genet Evol. 2020;80:e104173.

[10] Song R, Zhang D, Gao JW, et al. Characterization of the complete mitochondrial genome of *Brentisentis yangtzensis* Yu & Wu, 1989 (Acanthocephala, Illiosentidae). ZooKeys 2019b;861:1–14.

[11] Zhao TY, Yang RJ, Lü L, et al. Phylomitogenomic analyses provided further evidence for the resurrection of the family Pseudoacanthocephalidae (Acanthocephala: Echinorhynchida). Animals. 2023;13:e1256.

[12] Steinauer ML, Nickol BB, Broughton R, et al. First sequenced mitochondrial genome from the phylum Acanthocephala (*Leptorhynchoides thecatus*) and its phylogenetic position within Metazoa. J Mol Evol. 2005;60:706–15.

[13] Gao JW, Yuan XP, Wu H, *et al*. Mitochondrial phylogenomics of Acanthocephala: nucleotide alignments produce long-branch attraction artefacts. Parasite Vector. 2022;15:e376.

[14] Muhammad N, Suleman, Khan MS., et al. Characterization of the complete mitogenome of *Centrorhynchus clitorideus* (Meyer, 1931) (Palaeacanthocephala: Centrorhynchidae), the largest mitochondrial genome in Acanthocephala, and its phylogenetic implications. Mol Biochem Parasitol. 2020;237:e111274.

[15] Muhammad N, Suleman, Ma J, *et al*. Characterization of the complete mitochondrial genome of *Centrorhynchus milvus* (Acanthocephala: Polymorphida) and its phylogenetic implications. Infect Genet Evol. 2019;75:e103946.

[16] Gazi M, Kim J, García-Varela M, et al. Mitogenomic phylogeny of Acanthocephala reveals novel class relationships. Zool Scr. 2016;45:437–54.

[17] Muhammad N, Suleman, Ahmad MS, et al. Mitochondrial DNA dataset suggest that the genus *Sphaerirostris* Golvan, 1956 is a synonym of the genus *Centrorhynchus* Lühe, 1911. Parasitology. 2020;147:1149–57.

[18] Muhammad N, Suleman, Ma J, et al. Characterization of the complete mitochondrial genome of *Sphaerirostris picae* (Rudolphi, 1819) (Acanthocephala: Centrorhynchidae), representative of the genus *Sphaerirostris*. Parasitol Res. 2019;118:2213–21.

[19] Sarwar H, Zhao WT, Kibet CJ, et al. Morphological and complete mitogenomic characterisation of the acanthocephalan *Polymorphus minutus* infecting the duck *Anas platyrhynchos*. Folia Parasit. 2021;68:e15.

[20] Gazi M, Kim J, Park JK. The complete mitochondrial genome sequence of *Southwellina hispida* supports monophyly of Palaeacanthocephala (Acanthocephala: Polymorphida). Parasitol Int. 2015;64:64–8.

[21] Li DX, Yang RJ, Chen HX, et al. Characterization of the complete mitochondrial genomes of the zoonotic parasites *Bolbosoma nipponicum* and *Corynosoma villosum* (Acanthocephala: Polymorphida) and the molecular phylogeny of the order Polymorphida. Parasitology 2024;151:45–57.

[22] Garcia-Gallego A, Raga JA, Fraija-Fernandez N, *et al*. Temporal and geographical changes in the intestinal helminth fauna of striped dolphins, *Stenella coeruleoalba*, in the Western Mediterranean: a long-term analysis (1982–2016). Front Mar Sci. 2023;10:1–17.
