# Supplementary material for: Integrative taxonomy of the genus Pseudoacanthocephalus (Acanthocephala: Echinorhynchida) in China, with the description of two new species and the characterization of the mitochondrial genomes of Pseudoacanthocephalus sichuanensis sp. n. and Pseudoacanthocephalus nguyenthileae
Source: Parasit Vectors. 2024 Dec 27;17:541. doi: 10.1186/s13071-024-06528-7 (PMC11681651; doi:10.1186/s13071-024-06528-7)
Supplement: Supplementary file 6 — Additional file 6: Table S3. The partitioning schemes and the optimal amino acid substitution model selected for each combination of partition for the BI and ML inference. [file 13071_2024_6528_MOESM6_ESM.docx]

**Table S3.** The partitioning schemes and the optimal amino acid substitution model selected for each combination of partition for the BI and ML inference.

| Subset | Best model (BI/ML) | Partitioning schemes |
| --- | --- | --- |
| 1 | JTT+F+I+G4/mtInv+F+R5 | *atp6*, *cox3*, *nad2*, *nad3*, *nad4L*, *nad4*, *nad5*, *nad6* |
| 2 | WAG+F+I+G4/mtInv+F+R5 | *cox1*, *cox2*, *cytb*, *nad1* |
