# Supplementary material for: Integrative taxonomy of the genus Pseudoacanthocephalus (Acanthocephala: Echinorhynchida) in China, with the description of two new species and the characterization of the mitochondrial genomes of Pseudoacanthocephalus sichuanensis sp. n. and Pseudoacanthocephalus nguyenthileae
Source: Parasit Vectors. 2024 Dec 27;17:541. doi: 10.1186/s13071-024-06528-7 (PMC11681651; doi:10.1186/s13071-024-06528-7)
Supplement: Supplementary file 8 — Additional file 8: Table S5. Annotations and gene organization of P. nguyenthileae. Positive number in the “Gap or overlap” column indicates the length of intergenic sequence, and the negative number indicates the length (absolute number) that adjacent genes overlap (negative sign). [file 13071_2024_6528_MOESM8_ESM.docx]

**Table S5.** Annotations and gene organization of *P. nguyenthileae*. Positive number in the “Gap or overlap” column indicates the length of intergenic sequence, and the negative number indicates the length (absolute number) that adjacent genes overlap (negative sign). The forward strand is marked as “+” and the reverse strand as “-”.

| Gene | Type | Start | End | Length | Start Codon | Stop Codon | Anticodon | Strand | Gap or overlap |
| --- | --- | --- | --- | --- | --- | --- | --- | --- | --- |
| *cox1* | CDS | 1 | 1539 | 1539 | GTG | TAG |  | + | -2 |
| tRNA-Gly (G) | tRNA | 1538 | 1589 | 52 |  |  | UCC | + | -8 |
| tRNA-Gln (Q) | tRNA | 1582 | 1638 | 57 |  |  | UUG | + | -2 |
| tRNA-Tyr (Y) | tRNA | 1637 | 1688 | 52 |  |  | GUA | + | 0 |
| *rrnL* | rRNA | 1689 | 2589 | 901 |  |  |  | + | 9 |
| tRNA-Leu1 (L1) | tRNA | 2599 | 2668 | 70 |  |  | UAG | + | 1 |
| *nad6* | CDS | 2670 | 3138 | 469 | ATG | T |  | + | 4 |
| tRNA-Asp (D) | tRNA | 3143 | 3200 | 58 |  |  | GUC | + | 8 |
| *atp6* | CDS | 3209 | 3767 | 559 | ATG | T |  | + | 10 |
| *nad3* | CDS | 3778 | 4113 | 336 | ATG | TAA |  | + | 3 |
| tRNA-Trp (W) | tRNA | 4117 | 4176 | 60 |  |  | UCA | + | 0 |
| NCR1 | Non-coding region | 4177 | 4378 | 202 |  |  |  | + | 0 |
| tRNA-Val (V) | tRNA | 4379 | 4433 | 55 |  |  | UAC | + | -8 |
| tRNA-Lys (K) | tRNA | 4426 | 4475 | 50 |  |  | UUU | + | 3 |
| tRNA-Glu (K) | tRNA | 4479 | 4530 | 52 |  |  | UUC | + | -1 |
| tRNA-Thr (E) | tRNA | 4530 | 4591 | 62 |  |  | UGU | + | -11 |
| tRNA-Ser2 (S2) | tRNA | 4581 | 4630 | 50 |  |  | UGA | + | 0 |
| *nad4L* | CDS | 4631 | 4870 | 240 | GTG | TAG |  | + | 2 |
| *nad4* | CDS | 4873 | 6100 | 1228 | TTG | T |  | + | 3 |
| tRNA-His (H) | tRNA | 6104 | 6153 | 50 |  |  | GUG | + | 5 |
| *nad5* | CDS | 6159 | 7782 | 1624 | GTG | T |  | + | -3 |
| tRNA-Leu2 (L2) | tRNA | 7780 | 7841 | 62 |  |  | UAA | + | -14 |
| tRNA-Pro (P) | tRNA | 7828 | 7888 | 61 |  |  | UGG | + | -3 |
| *cytb* | CDS | 7886 | 9007 | 1122 | TTG | TAG |  | + | -1 |
| *nad1* | CDS | 9007 | 9880 | 874 | GTG | T |  | + | 5 |
| tRNA-Ile (I) | tRNA | 9886 | 9942 | 57 |  |  | GAU | + | 0 |
| NCR2 | Non-coding region | 9943 | 10560 | 618 |  |  |  |  | 0 |
| tRNA-Met (M) | tRNA | 10561 | 10614 | 54 |  |  | CAU | + | 0 |
| *rrnS* | rRNA | 10615 | 11182 | 568 |  |  |  | + | -2 |
| tRNA-Phe (F) | tRNA | 11181 | 11235 | 55 |  |  | GAA | + | -2 |
| *cox2* | CDS | 11234 | 11852 | 619 | ATG | T |  | + | 2 |
| tRNA-Cys (C) | tRNA | 11855 | 11908 | 54 |  |  | GCA | + | 7 |
| *cox3* | CDS | 11916 | 12617 | 702 | GTG | TAA |  | + | -1 |
| tRNA-Ala (A) | tRNA | 12617 | 12672 | 56 |  |  | UGC | + | -3 |
| tRNA-Arg (R) | tRNA | 12670 | 12720 | 51 |  |  | UCG | + | -9 |
| tRNA-Asn (N) | tRNA | 12712 | 12766 | 55 |  |  | GUU | + | -13 |
| tRNA-Ser1 (S1) | tRNA | 12754 | 12806 | 53 |  |  | ACU | + | 0 |
| *nad2* | CDS | 12807 | 13700 | 894 | GTG | TAA |  | + | 1 |
