# Supplementary material for: Integrative taxonomy of the genus Pseudoacanthocephalus (Acanthocephala: Echinorhynchida) in China, with the description of two new species and the characterization of the mitochondrial genomes of Pseudoacanthocephalus sichuanensis sp. n. and Pseudoacanthocephalus nguyenthileae
Source: Parasit Vectors. 2024 Dec 27;17:541. doi: 10.1186/s13071-024-06528-7 (PMC11681651; doi:10.1186/s13071-024-06528-7)
Supplement: Supplementary file 9 — Additional file 9: Table S6. Base composition and skewness of Pseudoacanthocephalus sichuanensis sp. n. and P. nguyenthileae. [file 13071_2024_6528_MOESM9_ESM.docx]

**Table S6.** Base composition and skewness of *Pseudoacanthocephalus sichuanensis* sp. n. and *P. nguyenthileae*.

| Location/Species | A (%) | T (%) | C (%) | G (%) | A+T (%) | AT skew | GC skew | Total |
| --- | --- | --- | --- | --- | --- | --- | --- | --- |
| ***P. sichuanensis* sp. n.** |  |  |  |  |  |  |  |  |
| Whole mitochondrial genome | 21.23 | 35.56 | 10.21 | 33 | 56.79 | -0.25 | 0.53 | 15812 |
| Protein coding genes (PCGs) | 17.43 | 37.48 | 10.15 | 34.94 | 54.91 | -0.37 | 0.55 | 10179 |
| Condon position |  |  |  |  |  |  |  |  |
| 1st codon | 20.43 | 30.64 | 9.86 | 39.06 | 51.07 | -0.20 | 0.60 | 3397 |
| 2nd codon | 13.95 | 47.04 | 11.5 | 27.51 | 60.98 | -0.54 | 0.41 | 3391 |
| 3rd codon | 17.9 | 34.77 | 9.08 | 38.25 | 52.67 | -0.32 | 0.62 | 3391 |
| tRNAs | 23.71 | 35.07 | 10.95 | 30.27 | 58.79 | -0.19 | 0.47 | 1206 |
| rRNAs | 26.89 | 33.92 | 11.69 | 27.5 | 60.81 | -0.12 | 0.40 | 1480 |
| *rrnL* | 26.76 | 35.21 | 10.21 | 27.82 | 61.97 | -0.14 | 0.46 | 568 |
| *rrnS* | 26.97 | 33.11 | 12.61 | 27.3 | 60.09 | -0.10 | 0.37 | 912 |
| Non-coding region 1 | 30.15 | 31.03 | 4.43 | 34.4 | 61.17 | -0.01 | 0.77 | 2282 |
| Non-coding region 2 | 31.85 | 26.15 | 26.77 | 15.23 | 58.00 | 0.10 | -0.27 | 650 |
| ***P. nguyenthileae*** |  |  |  |  |  |  |  |  |
| Whole mitochondrial genome | 19.79 | 36.51 | 10.66 | 33.03 | 56.3 | -0.30 | 0.51 | 13701 |
| Protein coding genes (PCGs) | 17.51 | 37.65 | 9.79 | 35.05 | 55.16 | -0.37 | 0.56 | 10206 |
| Condon position |  |  |  |  |  |  |  |  |
| 1st codon | 20.70 | 30.48 | 9.81 | 39.02 | 51.17 | -0.19 | 0.60 | 3406 |
| 2nd codon | 13.88 | 47.40 | 11.11 | 27.61 | 61.28 | -0.55 | 0.43 | 3401 |
| 3rd codon | 17.94 | 35.14 | 8.44 | 38.49 | 53.07 | -0.32 | 0.64 | 3401 |
| tRNAs | 24.80 | 35.15 | 10.44 | 29.61 | 59.95 | -0.17 | 0.48 | 1226 |
| rRNAs | 27.43 | 34.58 | 10.96 | 27.03 | 62.01 | -0.12 | 0.42 | 1469 |
| *rrnL* | 26.97 | 34.52 | 11.54 | 26.97 | 61.49 | -0.12 | 0.40 | 901 |
| *rrnS* | 28.17 | 34.68 | 10.04 | 27.11 | 62.85 | -0.10 | 0.46 | 568 |
| Non-coding region 1 | 27.72 | 31.19 | 1.49 | 39.6 | 58.91 | -0.06 | 0.93 | 202 |
| Non-coding region 2 | 28.08 | 25.81 | 28.41 | 17.69 | 53.9 | 0.04 | -0.23 | 616 |
